# Towards a dynamic photosynthesis model to guide yield improvement in C4 crops

**DOI:** 10.1111/tpj.15365

**Published:** 2021-08-06

**Authors:** Yu Wang, Kher Xing Chan, Stephen P. Long

**Affiliations:** ^1^ Carl R Woese Institute for Genomic Biology University of Illinois at Urbana‐Champaign Urbana IL 61801 USA; ^2^ DOE Center for Advanced Bioenergy and Bioproducts Innovation University of Illinois at Urbana‐Champaign Urbana IL 61801 USA; ^3^ Departments of Plant Biology and of Crop Sciences University of Illinois at Urbana‐Champaign Urbana IL 61801 USA; ^4^ Lancaster Environment Centre Lancaster University Lancaster LA1 4YQ UK

**Keywords:** photosynthetic induction, C4 photosynthesis, *Zea mays*, *Sorghum bicolor*, *Saccharum officinarum*, mathematical model, Rubisco activase, PPDK regulatory protein, stomatal conductance

## Abstract

The most productive C4 food and biofuel crops, such as *Saccharum officinarum* (sugarcane), *Sorghum bicolor* (sorghum) and *Zea mays* (maize), all use NADP‐ME‐type C4 photosynthesis. Despite high productivities, these crops fall well short of the theoretical maximum solar conversion efficiency of 6%. Understanding the basis of these inefficiencies is key for bioengineering and breeding strategies to increase the sustainable productivity of these major C4 crops. Photosynthesis is studied predominantly at steady state in saturating light. In field stands of these crops light is continually changing, and often with rapid fluctuations. Although light may change in a second, the adjustment of photosynthesis may take many minutes, leading to inefficiencies. We measured the rates of CO_2_ uptake and stomatal conductance of maize, sorghum and sugarcane under fluctuating light regimes. The gas exchange results were combined with a new dynamic photosynthesis model to infer the limiting factors under non‐steady‐state conditions. The dynamic photosynthesis model was developed from an existing C4 metabolic model for maize and extended to include: (i) post‐translational regulation of key photosynthetic enzymes and their temperature responses; (ii) dynamic stomatal conductance; and (iii) leaf energy balance. Testing the model outputs against measured rates of leaf CO_2_ uptake and stomatal conductance in the three C4 crops indicated that Rubisco activase, the pyruvate phosphate dikinase regulatory protein and stomatal conductance are the major limitations to the efficiency of NADP‐ME‐type C4 photosynthesis during dark‐to‐high light transitions. We propose that the level of influence of these limiting factors make them targets for bioengineering the improved photosynthetic efficiency of these key crops.

## Significance Statement

Despite high productivities, the photosynthetic efficiency of C4 crops is well below the theoretical value. Analyses of limitations have focused on constant conditions, but fluctuating light prevails in crop canopies in the field. A combined dynamic metabolic, stomatal and leaf microclimate model is developed and parameterized for maize, sorghum and sugarcane. The model shows that stomatal opening and the proteins activating Rubisco and PPDK are the major limitations to photosynthesis under fluctuating light. The findings indicate new targets for increasing the productivity of these key crops.

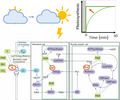

## INTRODUCTION

Increasing crop genetic yield potential during the Green Revolution proved critical both to global food security and reducing the land‐use change that would otherwise have been necessary to support the growing world population. The biggest contribution from this period was from new genotypes of major grain crops, which had greatly improved harvest indices and, coupled with improved agronomy, provided increased yield (FAO, IFAD and UNICEF, 2018, 2020). However, after rapid increases in yield over the 45 years from 1960, the growth in productivity of the world’s major crops is stagnating (Ray et al., 2012). With a forecast 60% increase in global demand for primary foodstuffs by mid‐century there is an urgent need to find new means to sustainably increase the productivity of these key crops (FAO, IFAD and UNICEF, 2020).

In parallel, a second global challenge is how to provide sustainable sources of energy and bioproducts to meet increasing societal pressures to achieve zero net glasshouse gas emissions. Displacing 55–70% of the petroleum demand in the USA with cellulosic biofuels would require about 10^9^ Mg of biomass (Langholtz et al., 2016; Robertson et al., 2017). *Saccharum officinarum* (sugarcane) and *Zea mays* (maize) are the largest current sources of biofuels, whereas *Sorghum bicolor* (sweet and fiber sorghum) and the sugarcane‐derivative fiber crop, energycane, have emerged as major potential bioenergy and bioproduct feedstocks (Crow et al., 2020; Gautam et al., 2020; Jaiswal et al., 2017; Long et al., 2015; Parajuli et al., 2020). All belong to the monophyletic grass tribe Andropogoneae and all predominantly use the C4 NADP‐ME metabolic pathway (Bianconi et al., 2019). Increasing crop photosynthetic efficiency is one means to meet the need to increase the yields of food, biofuels and bioproduct crops and so avoid a need to bring more land into agriculture.

The yield potential of a given genotype at a given location is the product of the incident photosynthetically active radiation over the growing season, the efficiency of the crop in intercepting that radiation (ɛ_i_), the efficiency of the conversion of intercepted radiation into plant mass (ɛ_c_) and the efficiency of partitioning that mass into the harvested product (ɛ_p_), also termed harvest index (Zhu et al., 2010); see Table 1 for a list of abbreviations, their definitions and units. Plant breeding has optimized ɛ_i_ and ɛ_p_ to the point where there is little opportunity for further improvement of these efficiencies in the major crops (Long et al., 2019; Zhu et al., 2010). Harvest indices are now as high as 0.6 in modern crop cultivars, with little scope for further improvement in this trait (Evans, 1997; Murchie et al., 2009; Zhu et al., 2010). Under optimal conditions the ɛ_p_ of maize hybrids is about 0.52, which has remained unchanged for the last two decades (Di Matteo et al., 2016), whereas sugarcane has an already high ɛ_p_ of 0.8, as the majority of the plant is the harvested product (i.e. stem) (Waclawovsky et al., 2010). By contrast, the third factor ɛ_c_ (also known as light‐use efficiency) of C3 and C4 crops, governed by photosynthesis, falls well below its theoretical maximum (Zhu et al., 2008). Theoretical analysis and genetic engineering have shown considerable potential to increase photosynthetic efficiency in both C3 and C4 crops (Kromdijk et al., 2016; Long et al., 2019; López‐Calcagno et al., 2020; Murchie et al., 2009; Salesse‐Smith et al., 2018; South et al., 2019; Yoon et al., 2020). Although increasing the stress tolerance of crops is another important route to increasing productivity, experience shows that increasing genetic yield potential can address both of these factors, thereby increasing the average yields achieved under both optimal and stress conditions (Wu et al., 2019). For example, a detailed analysis of progressive gains in yield potential through soybean breeding have resulted in achieved yield increases in years with both good and suboptimal production conditions (Koester et al., 2014).

Mathematical modeling of the photosynthetic process and the application of optimization methods have proven valuable in identifying targets for the bioengineering of increased efficiency and sustainability (Wang et al., 2014; Zhu et al., 2007, 2004). They have led to proof‐of‐concept improvements in productivity in replicated field trials with genetically engineered *Nicotiana* *tabacum* (tobacco) (Kromdijk et al., 2016; López‐Calcagno et al., 2020; South et al., 2019). However, these models have focused largely on the steady state and often on light‐saturated conditions, as have most measurements and analyses of the limitations of leaf photosynthesis.

In the field, however, leaves are rarely in steady state, and are subject to frequent fluctuations in light intensity. There has been a growing awareness of the need to address photosynthetic efficiency in fluctuating light conditions, as well as under constant light (Acevedo‐Siaca et al., 2020b; De Souza et al., 2020; Deans et al., 2019; Hubbart et al., 2012; McAusland and Murchie, 2020; McAusland et al., 2016; Murchie and Ruban, 2020).

A great deal of progress has been made towards understanding the dynamic response to light in C3 plants. The non‐steady‐state photosynthetic rate of C3 plants under fluctuating light is mainly affected by the speed of the following responses: the relaxation of non‐photochemical quenching; the activation and de‐activation of Rubisco; the activation of ribulose 1,5‐bisphosphate (RuBP) regeneration enzymes; metabolite pool sizes; mesophyll conductance and stomatal conductance (Deans et al., 2019; Kaiser et al., 2016; Mott and Woodrow, 2000; Pearcy, 1994; Slattery et al., 2018). However, the major limitations vary with species (Acevedo‐Siaca et al., 2020; De Souza et al., 2020; McAusland et al., 2016; Taylor and Long, 2017).

Dynamic photosynthetic models infer that faster responses of photosynthesis to light fluctuations within canopies could increase the productivity of C3 crops by 13–32% (Taylor and Long, 2017; Wang et al., 2020; Zhu et al., 2004). A bioengineered increase in the speed of the relaxation of non‐photochemical quenching, upon transition from high light to low light, significantly increased the quantum yield of photosynthesis in tobacco grown under fluctuating light, and also resulted in an approximately 20% increase in productivity in replicated field trials (Kromdijk et al., 2016).

Few experimental studies of photosynthesis under fluctuating light conditions in C4 plants have been published, however. The loss of productivity in fluctuating light, relative to constant light, in two C4 species (*Amaranthus* *caudatus* and *Setaria macrostachya*) was greater than in two C3 species (*Celosia argentea* and *Triticum aestivum*), implying a greater impact on C4 plants (Kubásek et al., 2013). Aside from this experiment, two early sets of gas‐exchange measurements suggested that stomatal conductance is not a limiting factor during photosynthetic induction in maize (Furbank and Walker, 1985; Usuda and Edwards, 1984).

Energy‐use efficiency limitations of C4 photosynthesis under steady‐state conditions have been analyzed via a number of empirical analyses and biochemical models (Bellasio and Griffiths, 2014; Laisk and Edwards, 2000; Wang et al., 2014a,b; Yin and Struik, 2018; Yin and Struik, 2021). However, no mechanistic modeling studies have analyzed the limitations of non‐steady‐state C4 photosynthesis. Here, we developed a dynamic model to predict the potential limitations within C4 photosynthesis in fluctuating light to suggest feasible targets to improve the energy‐use efficiency of C4 crops. As the major food and fiber C4 crops maize, sorghum, sugarcane and *Miscanthus* predominantly use the NADP‐ME form of C4 photosynthesis, we previously developed a kinetic metabolic model of this form of C4 photosynthesis. It represented all discrete metabolic reactions of photosynthesis within the different cellular compartments of a C4 leaf, and transfer between the compartments, as a system of ordinary differential equations (ODEs). Each individual reaction and transfer between compartments is described using either enzyme kinetics or metabolite diffusion kinetics (Wang et al., 2014). This metabolic model was used to predict the limitations of C4 photosynthesis under steady‐state lighting at 25°C.

To predict limitations under non‐steady‐state conditions, this metabolic model was extended to capture the key factors affecting non‐steady‐state photosynthesis during transitions from low light to high light, and vice versa. Specifically, we included the post‐translational regulation of key photosynthetic enzymes, temperature responses of the enzyme activities, dynamic stomatal conductance and leaf energy balance. We then parameterized the model using gas‐exchange data for the three most widely grown NADP‐ME C4 crops: maize, sorghum and sugarcane. From this, the major factors limiting photosynthesis during transitions from the dark to high light for these crops were predicted to be Rubisco activase (Rca), the pyruvate, phosphate dikinase (PPDK) regulatory protein and stomatal conductance.

## RESULTS

### Factors influencing induction of C4 photosynthesis in transitions from dark to high light

**Figure 1:**
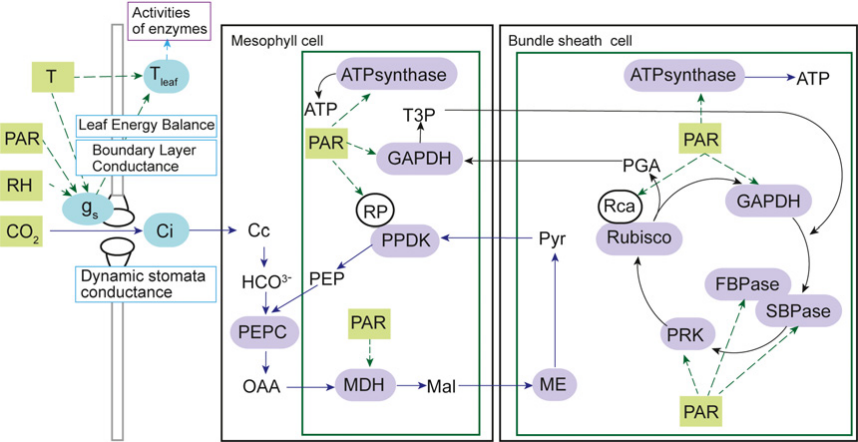
Metabolic model schematic of C4 photosynthesis. The model includes all metabolites and enzymes of photosynthetic carbon metabolism, as detailed previously (Wang et al., 2014). Here, only enzymes that are light modulated and therefore modified in this new dynamic model are indicated. Green rectangles are driving environmental variables affecting enzyme activities (purple) and stomatal conductance. Blue blocks are state variables calculated from leaf energy balance for *T*_leaf_, dynamic stomatal response model for stomatal conductance (*g_s_*), and from the external [CO_2_], *g_s_* and predicted leaf CO_2_ uptake rate for *C_i_*.

**Figure 2:**
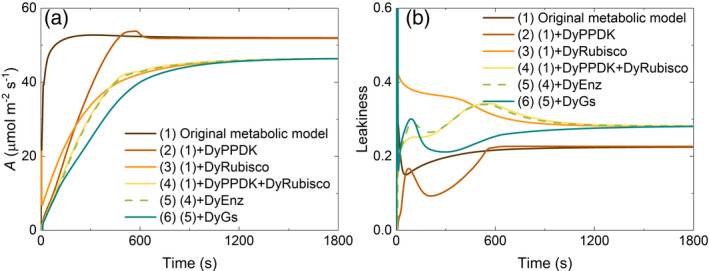
Simulated induction of: (a) leaf CO_2_ uptake (*A*); and (b) bundle‐sheath leakiness (ϕ), with various dynamic regulation settings. Scenario 1 uses the original metabolic model (Wang et al., 2014), which assumes steady‐state light activated enzyme activity and stomatal conductance from time zero, that is, throughout. In scenarios 2 and 3, DyPPDK and DyRubisco are added to the steady‐state model of scenario 1 to model the induction responses of pyruvate phosphate dikinase (PPDK) and Rubisco, respectively, regulated by the action of the PPDK regulatory protein (PDRP) and by the action of Rubisco activase (Rca) for Rubisco, respectively. Scenario 4 combines scenarios 2 and 3, and scenario 5 includes all light‐regulated enzymes. Scenario 6 superimposes stomatal opening on scenario 5. The induction simulates transfer from darkness to full sunlight: 1800 µmol m^−2^ sec^−1^. The input parameters are those listed in Table 2: ‘Original values’.

The dynamic model presented here extends a previous C4 metabolic model (Wang et al., 2014a,b) to include post‐translational regulation, temperature responses of enzymes, dynamic stomatal conductance and leaf energy balance (Figure 1). The model was built by superimposing the dynamic regulation of enzyme activation and stomatal conductance on the metabolic NADP‐ME C4 leaf photosynthesis model of Wang et al. (2014). This was initially parameterized from the literature (Table 2). During induction some C4 metabolic pools, in particular malate in the bundle sheath cytoplasm, rise to very high concentrations (Leegood, 1997). To assess the role of this accumulation of photosynthetic metabolites during induction, the model was first run assuming that all enzymes were fully activated and the stomata open. Termed scenario 1, this resulted in a rapid induction to near steady state within 120 sec (Figure 2a). The major limitation over this period was the time taken for C4 metabolites to accumulate and approach steady state, lagging C3 metabolites (Figure S1). Leakiness (ϕ), that is the proportion of CO_2_ released by decarboxylation in the bundle sheath that diffused back to the mesophyll, reached a minimum at 30 sec, gradually climbing to a steady‐state value of approximately 0.22 at approximately 600 sec, indicating that the flux through the C4 cycle continued to limit photosynthesis (Figure 2b). This limitation was affected by the activity of mutase and enolase, the enzymes that convert PGA to PEP. Increasing the maximum activity of mutase and enolase accelerated induction in scenario 1 (Figure S2).

In scenario 2, the regulation of PPDK by its regulatory protein (PDRP) substantially slowed the rate of induction (d*A*/d*t*) of the CO_2_ assimilation rate (*A*) (Figure 2a) by limiting the synthesis of phosphoenolpyruvate (PEP), and thus lowering the predicted ϕ (Figure 2b). In scenario 3, Rubisco regulation alone is added and resulted in a similar decrease in the rate of induction of (d*A*/d*t*) to that of scenario 2. It reduced the final steady‐state level of *A*, as a greater proportion of Rubisco now remained inactive (Figure 2a). As would be expected, in contrast to scenario 2, leakiness is high in scenario 3 throughout induction, as the C4 cycle is delivering CO_2_ to the bundle sheath, but Rubisco is not fully activated and so is less able to use the CO_2_ being released by malate decarboxylation (Figure 2b). Combining the activation of both PPDK and Rubisco to give scenario 4 results in a yet slower rate of induction (Figure 2a), but the closer coordination of the activation of the two enzymes results in less bundle‐sheath leakiness during induction. The simulated leakiness increases for the first 600 sec and then declines to a steady‐state value of approximately 0.28 by 1200 sec, reflecting the predicted faster activation of PPDK than Rubisco (Figure 2b). The addition of dynamic control of the other light‐activated enzymes of photosynthetic carbon metabolism (Figure 1) in scenario 5 produces dynamic responses of *A* and ϕ that are almost identical to those of scenario 4 (Figure 2). Finally, superimposing the response of stomatal conductance (*g*_s_) in scenario 6 on the dynamics of *A* and intercellular CO_2_ concentration (*C*_i_) further slows the speed of induction, but dampens the bundle‐sheath leakage that would otherwise occur (Figure 2a,b).

**Figure 3:**
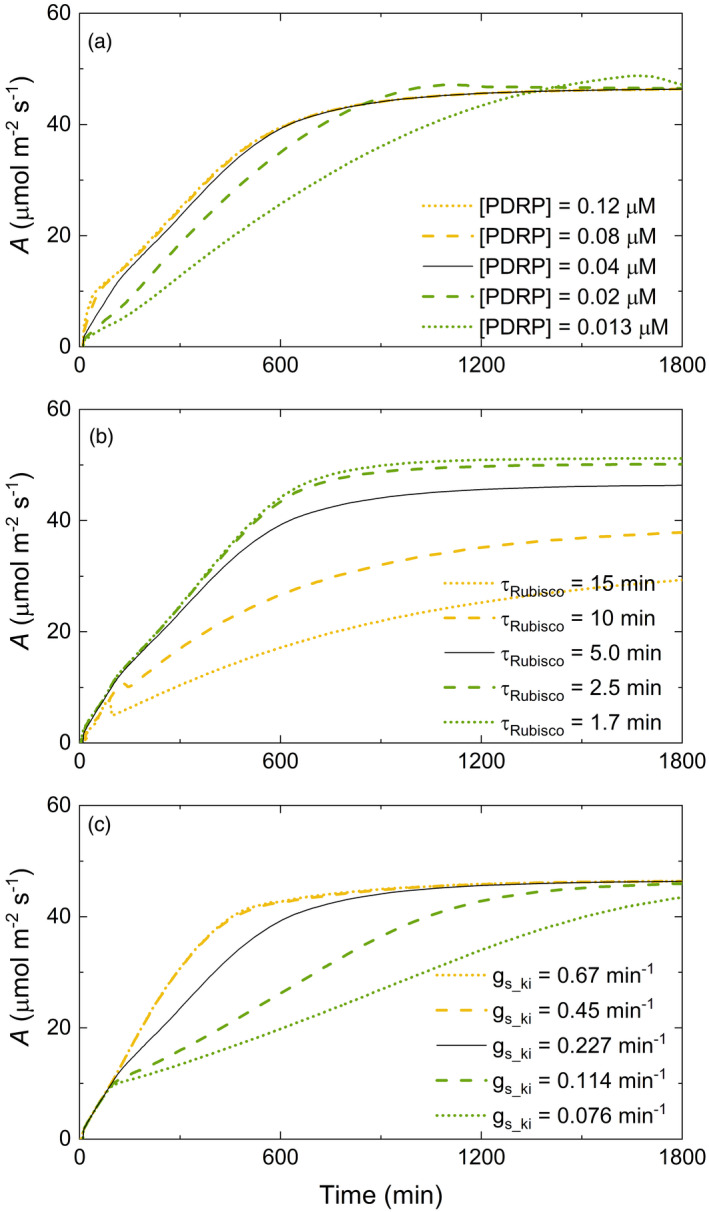
Simulated induction of leaf CO_2_ uptake (*A*) from dark to high light with variation in (a) the concentration of pyruvate phosphate dikinase regulatory protein (PDRP), (b) the time constant for the activation of Rubisco by Rubisco activase (τ_Rubisco_), and (c) the speed of stomatal opening (*g* *_s_ki_*); *k* *_i_* is the rate constant for increase in stomatal conductance. After dark adaptation, and at time 0 above, the light intensity was raised to 1800 µmol m^−2^ sec^−1^ to initiate induction. The input parameters and 'Original values' are listed in Table 2; symbols are defined in Table 1.

The model is shown to predict typical dynamic responses of *A* and *g*_s_, both with respect to pattern and magnitude during induction. The simulation predicts PPDK activation, Rubisco activation and stomatal dynamics as the major limitations, whereas the activation of other enzymes of carbon metabolism and metabolic pool size adjustment had little effect (Figure 3). The concentration of PDRP regulates the initial phase of the photosynthetic induction curve (Figure 3a), whereas the speed of Rubisco activation affects the later phase of the induction (Figure 3b). During the mid‐phase of induction, *g*_s_ is shown to limit *A* (Figure 3c).

### Measured photosynthetic induction of maize, sorghum and sugarcane

**Figure 4:**
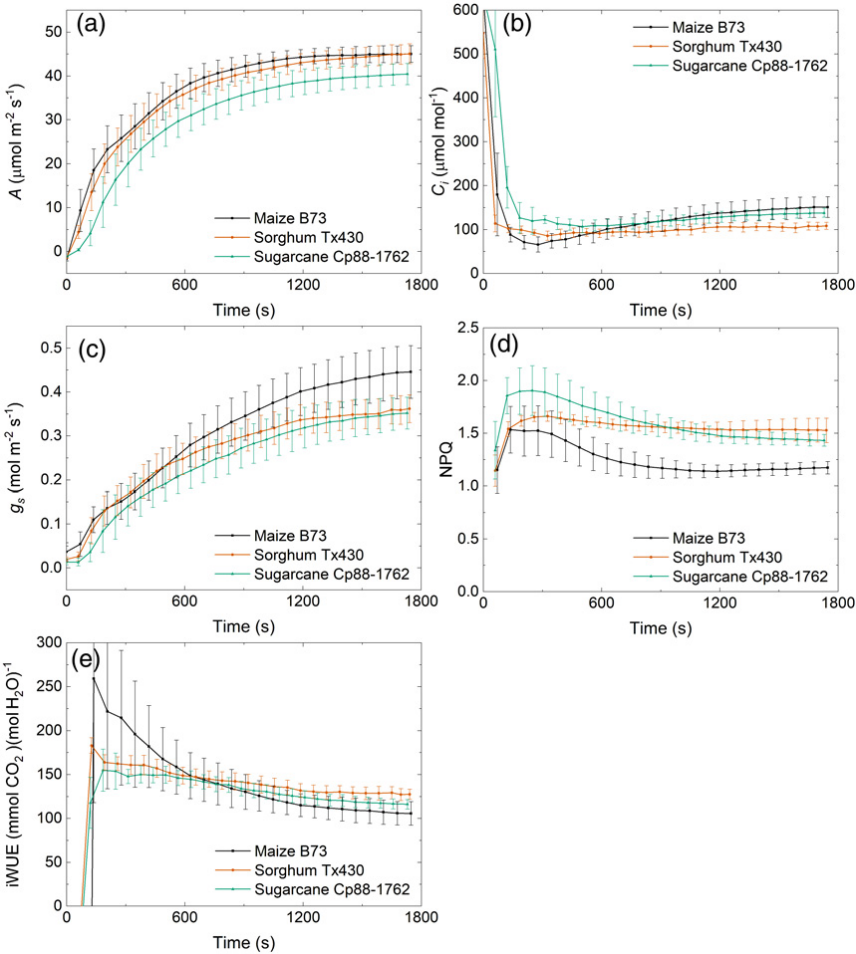
Measured gas exchange parameters upon transition from dark to high light (1800 µmol m^−2^ sec^−1^) after 30 min of dark adaptation: (a) leaf CO_2_ uptake rate (*A*); (b) intercellular CO_2_ concentration (*C_i_*); (c) stomata conductance (*g_s_*); (d) non‐photochemical quenching (NPQ); and (e) intrinsic water‐use efficiency (iWUE). Bars represent ±1 SEMs for six plants. Leaf CO_2_ uptake (*A*) of the youngest fully expanded leaf was measured on 30‐day‐old maize B73, sugarcane CP88‐1762 and sorghum Tx430 plants with a gas exchange system (LI‐6800, LI‐COR).

To further analyze the limitations for different C4 crop species, the steady‐state and dynamic gas exchange of three major C4 crops were measured, using one widely grown or well‐studied genotype for each species: maize B73, sugarcane CP88‐1762 and sorghum Tx430. During the transition from dark to high light, the CO_2_ assimilation rate of maize rose the fastest, followed by sorghum and then by sugarcane (Figure 4a). The time required to reach 50% of the steady‐state rate (IT50) for the three crops was 196, 237 and 316 sec, respectively. The average CO_2_ assimilation rate in the 30‐min induction was reduced by 17.7, 20.6 and 24.2% in maize, sorghum and sugarcane, respectively, compared with the steady‐state CO_2_ assimilation rate. However, maize had a slower rate of increase in *g*_s_ compared with sorghum and sugarcane (Figure 4c; Table 2). The *C*_i_ value dropped rapidly in the first approximately 100 sec, and then slowly increased to the steady‐state level. The lowest *C*_i_ values were 66, 86 and 107 µmol mol^−1^ in maize, sorghum and sugarcane, respectively, which are 53, 21 and 22% lower than their steady‐state *C*_i_ values (Figure 4b). The low *C*_i_ values would appear to be insufficient to fully saturate photosynthesis from about 180 to 600 sec after illumination began. Maize showed the highest intrinsic water‐use efficiency (iWUE) in the first 600 sec, whereas sorghum had the highest iWUE after 600 sec (Figure 4e). iWUE is the ratio of *A* to *g*_s_. Non‐photochemical quenching (NPQ) of the three species rose to a peak at approximately 60 sec and then declined to a steady state at approximately 600 sec, largely in concert with assimilation (Figure 4d).

### Model parameterization and validation

**Figure 5:**
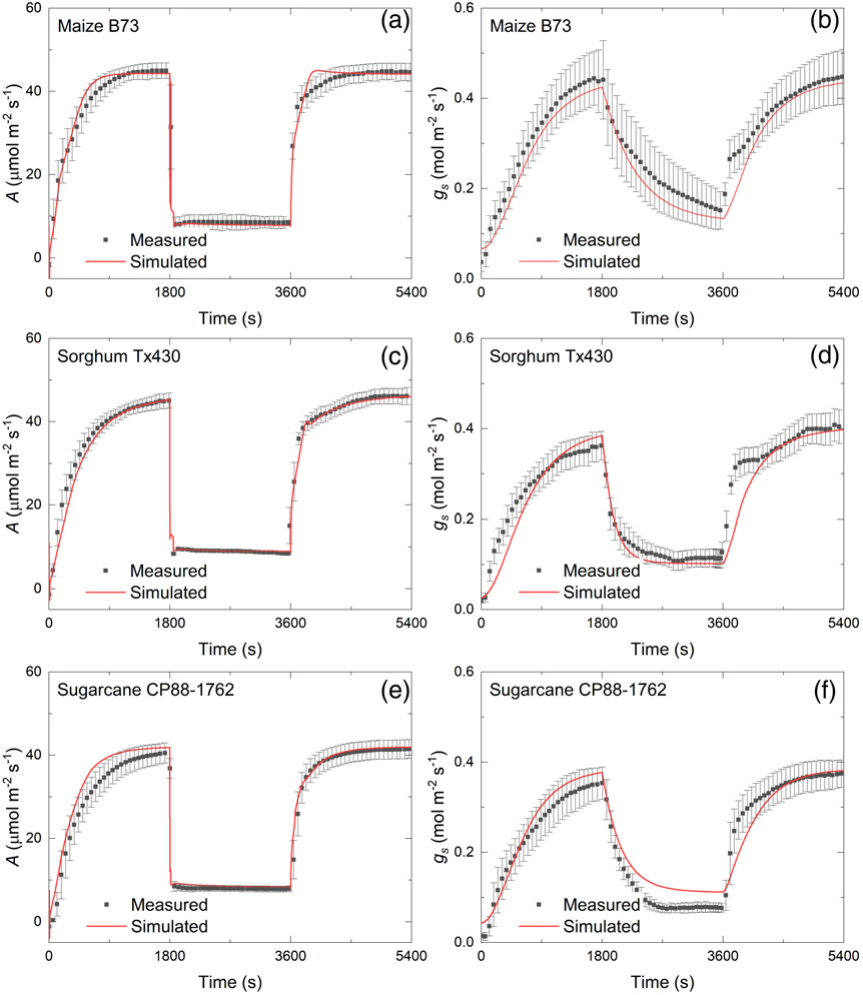
Simulated dynamic (a, c, e) photosynthesis (*A*) and (b, d, f) stomatal conductance (*g* *_s_*) under fluctuating light conditions, for (a, b) maize, (c, d) sorghum and (e, f) sugarcane. The simulation used the parameters of non‐steady‐state photosynthesis from scenario 6 in Figure 2, but calibrated to the measured steady‐state photosynthesis of Figure 4. The measurements were made on six replicate plants. Red lines are the simulated results and black dots are the means of the measured data ±1 SE. After 30 min of dark adaptation, the leaves underwent three light change steps beginning at time 0 in above Figures, with the light intensity set to 1800, 200 and 1800 µmol m^−2^ sec^−1^ for each 1800 sec (30 min) step. The input parameters are listed in Table 2.

Using the measured steady‐state and dynamic gas exchange data (Figures 4, 5 and Figure S6), we estimated the following photosynthetic parameters: maximum Rubisco activity (*V*_cmax_); maximum PEP carboxylase activity (*V*_pmax_); rate constants for stomatal conductance on dark‐to‐light and light‐to‐dark transitions (*k*_i_ and *k*_d_, respectively); time constant of Rubisco activation (τ_Rubisco_); mitochondrial respiration (*R*_d_); concentration of PDRP; and the slope and intercept for the model described by Ball et al. (1987) (Figures S3–S7; Table 2). Using these species‐specific parameters alone, the model was able to closely replicate the measured dynamics of *A* and *g*_s_ in all three C4 crops under fluctuating light (Figure 5). This consisted of 30 min of dark adaptation, followed by 30‐min intervals of high light, low light and high light again (Figure 5).

### Factors limiting the speed of photosynthetic induction

**Figure 6:**
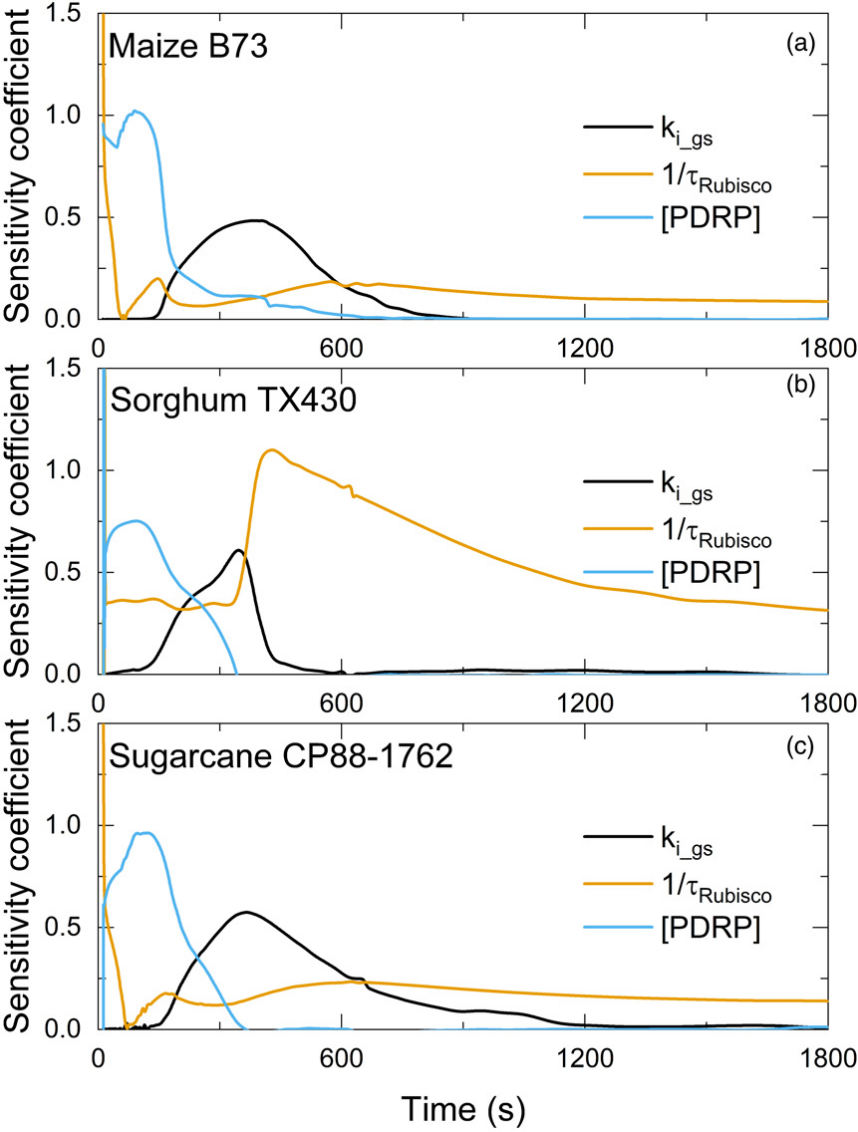
Simulated changes in the sensitivity coefficients of key parameters through photosynthetic induction for (a) maize, (b) sorghum and (c) sugarcane. After dark adaptation, light intensity was raised to 1800 µmol m^−2^ sec^−1^; time 0 above. To determine which steps in the system exert the strongest control on dynamic photosynthesis rate, a sensitivity analysis was performed by varying each parameter ±1%. Sensitivity coefficients are calculated as the ratio of change in the value of the parameter divided by change in leaf CO_2_ uptake rate (*A*), individually. If a 1% change in a parameter results in a 1% change in *A*, the sensitivity coefficient is 1, whereas if the change in *A* is zero, then the sensitivity coefficient is 0, meaning that no effect is exerted by that parameter. *k* *_i_gs_* is the time constant of stomata opening, τ_Rubisco_ is the time constant of Rubisco activation, and [PDRP] is the concentration of the pyruvate phosphate dikinase (PPDK) regulatory protein.

Sensitivity analysis of PDRP and Rca concentrations and the speed of the stomatal response indicated that all three limit the rate of photosynthetic induction upon transition from dark to light in the three C4 crops (Figure 6). However, the strength of each limitation varied between species and with time into induction. In maize B73, sensitivity analysis suggested that PDRP exerts the highest limitation in the first 200 sec of induction, followed by stomatal opening over the next 400 sec and then Rca slightly limits the remaining phase of induction (Figure 6a). In sorghum Tx430 and sugarcane CP88‐1762, the concentration of PDRP limits the rate of induction in the first 240 sec, a little longer than in maize (Figure 6b,c). Rca exerts more influence in sorghum, with a peak at around 420 sec (Figure 6b), whereas the Rca limitation in maize and sugarcane remains approximately constant over this time period (Figure 6a,c). Stomatal limitation was greater in maize and sugarcane compared with sorghum (Figure 6).

**Figure 7:**
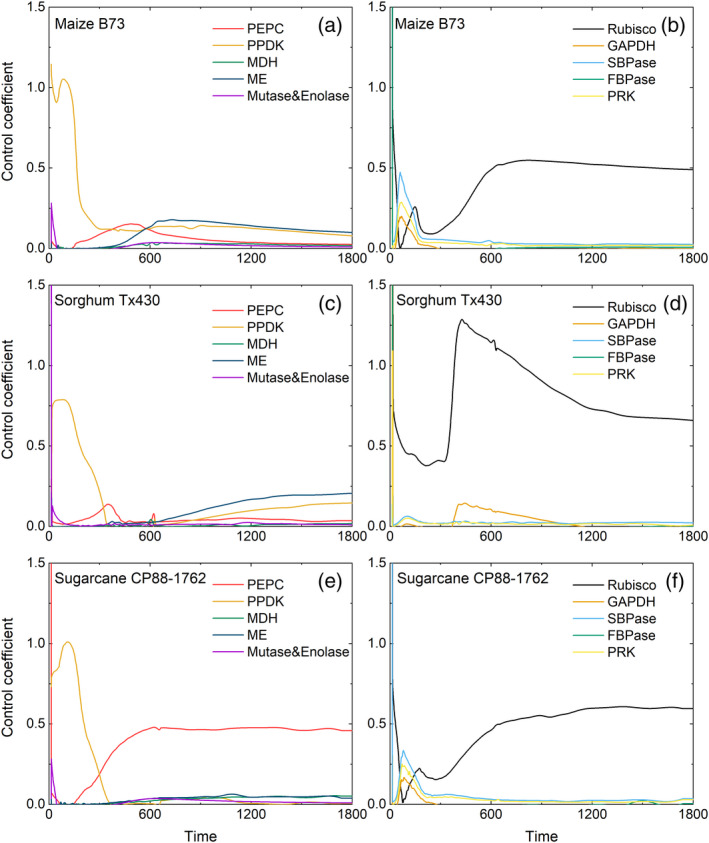
The control coefficient of the maximum activity of photosynthetic enzymes (*V*_max_) through time(s) of induction. After dark adaptation, the light intensity was raised to 1800 µmol m^−2^ sec^−1^. The photosynthetic enzymes shown here include: PEPC, phosphoenolpyruvate carboxylase; PPDK, pyruvate phosphate dikinase; MDH, malate dehydrogenase (NADP+); ME, NADP‐malic enzyme; mutase and enolase; Rubisco, ribulose‐bisphosphate carboxylase; GAPDH, glyceraldehyde‐3‐phosphate dehydrogenase (NADP+); SBPase, sedoheptulose‐bisphosphatase; FBPase, fructose‐bisphosphatase; PRK, phosphoribulokinase. (a, c, e) The predicted control coefficients of the C4 cycle enzymes. (b, d, f) The predicted control coefficients of the Calvin–Benson cycle enzymes.

In general, PPDK and Rubisco have high control coefficients in the first few minutes; whereas that of PPDK then declines, Rubisco continues to exert control through the mid and final stages of the induction. PEPC also has a high control coefficient from the middle of the induction in sugarcane (Figure 7e). PPDK and ME have some control in the later stage in maize and sorghum (Figure 7c). A control coefficient in the present context quantifies the relative influence of a single metabolic step on the rate of CO_2_ assimilation. A control coefficient of 1 indicates that the step has complete control and a control coefficient of zero indicates that the step has no control. Except for Rubisco, other light‐regulated enzymes of the Calvin–Benson cycle, including glyceraldehyde‐3‐phosphate dehydrogenase (GAPDH), sedoheptulose‐bisphosphatase (SBPase) and phosphoribulokinase (PRK), exerted only mild control in the first 150 sec of the induction (Figure 7b,d,f).

### Predicted CO_2_ leakiness (ϕ) during photosynthetic induction

**Figure 8:**
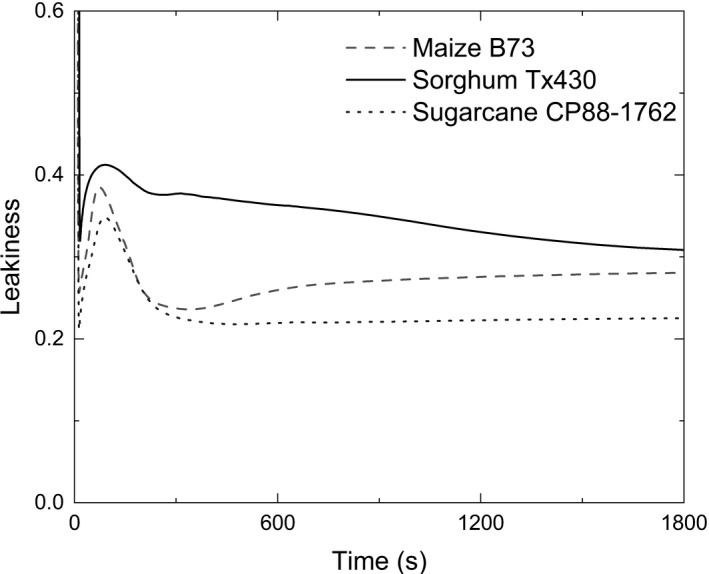
Simulated CO_2_ leakiness (ϕ) dynamics for maize, sorghum and sugarcane during photosynthetic induction following 30 min of dark adaptation. The light intensity was set to 1800 µmol m^−2^ sec^−1^. The input parameters are listed in Table 2: ‘Maize, sorghum and sugarcane’.

Predicted ϕ shows an increase as PPDK becomes activated and then declines as simulated Rubisco activity catches up for the three C4 crops. This suggested a loss of coordination between the C4 and Calvin–Benson cycles during induction (Figure 8). The simulated ϕ of sorghum declines more slowly than that of maize and sugarcane, because of the slower rate of Rubisco activation (Figure S4; Table 2).

## DISCUSSION

### **The energy**‐**use efficiency of C4 leaves is impacted under fluctuating light**

In this study, the average photosynthetic rate through the 30‐min induction was reduced by 18, 21 and 24% in maize, sorghum and sugarcane, respectively, as compared with the steady‐state photosynthetic rate (Figure 4a). This reduction has a very significant effect on the energy‐use efficiency and net carbon assimilation of crops in the field, as clouds, wind and the movement of the sun cause frequent light fluctuations within leaf canopies (Kaiser et al., 2018; Tanaka et al., 2019; Wang et al., 2020; Zhu et al., 2004). In sunny conditions, C4 crops have a higher light energy‐use efficiency compared with C3 crops as a result of the CO_2_ concentrating mechanism, which largely removes the energy cost of photorespiration (Zhu et al., 2010). However, C4 crops may be less resilient to fluctuating light levels, resulting in a decrease in productivity in dynamic light environments (Kubásek et al., 2013). This indicates a significant potential for yield improvement of C4 food and biofuel crops by engineering or breeding for improved speeds of adjustment to fluctuating light.

Including post‐translational regulation, the temperature response of enzyme activities, dynamic stomatal conductance and a leaf energy balance module, the model closely simulated the measured photosynthetic responses of these crops under fluctuating light (Figure 5), in contrast to the original metabolic model (Figure 2). This suggests that the model captured the key factors affecting the speed of induction upon light fluctuations (Figure 5). Using this model, we determined the factors influencing the speed of induction. With the species‐specific input parameters (Table 2), the model is able to predict the limiting factors under conditions of fluctuating light (Figures 6 and 7). This has identified potential targets for the improvement of energy‐use efficiency in maize, sorghum and sugarcane. Namely, the coordinated upregulation of Rca and the PPDK regulatory protein, as well as increased rates of stomatal adjustment.

### Limiting factors during photosynthetic induction

In C3 plants, the rate of photosynthetic induction is mainly limited by the activation of Rubisco, the activation of the enzymes affecting RuBP regeneration and the speed of stomata opening, with the major limitations varying between species (Acevedo‐Siaca et al., 2020; De Souza et al., 2020; McAusland et al., 2016; Taylor and Long, 2017). However, the limitations to C4 photosynthetic efficiency under fluctuating light have received little attention. Through a combination of model simulation and gas‐exchange experiment, we identified the following limiting factors in photosynthetic induction: (i) the accumulation of C4 photosynthetic intermediates to drive intercellular flux; (ii) the activation of PPDK; (iii) stomata opening; and (iv) the activation of Rubisco.

In our simulations, C4 cycle metabolites took longer to reach a steady state compared with Calvin–Benson cycle enzymes (Figure S1c,d), although the influence on the induction of photosynthesis was limited to the first 120 sec (Figure S1a). Also, accelerating the exchange of metabolites between the Calvin–Benson cycle and the C4 cycle, that is, increasing the activity of mutase and enolase, which catalyze the conversion of PGA to PEP, can further reduce the limitation of metabolites during this initial period of induction (Figure S2). We noted that mutase and enolase exerted higher control at the beginning of the induction and dropped to zero after about 60 sec, based on our control analysis for the three C4 crops (Figure 7). However, if the leaves experience a short‐term sun fleck, increased rates of photosynthetic metabolite accumulation would improve efficiency. The high concentration of C4 metabolites in NADP‐ME species at light saturation results in a slower decline in leaf CO_2_ uptake upon transitions from high light to shade, as the decarboxylation of malate continues to provide NADPH, compensating for the decline in NADPH from whole‐chain electron transport for a few minutes (Stitt and Zhu, 2014).

Our results infer that increasing the concentration of PDRP will increase the photosynthetic efficiency of these C4 plants under the fluctuating light conditions of field crop canopies. This is based on the simulation using the dynamic model developed in this study, which suggested that the concentration of PDRP is a major limitation during the first 180 sec of induction for maize, and for roughly the first 250 sec of induction for sorghum and sugarcane (Figure 6). It regulates both dark‐induced inactivation and light‐induced activation of PPDK by catalyzing the reversible phosphorylation of a threonine residue (Ashton et al., 1984; Budde et al., 1985; Burnell and Hatch, 1983, 1985; Chastain, 2010; Chastain et al., 2018). Although these studies elucidated the molecular mechanism for the activation of PPDK by PDRP, the direct effect of PDRP on photosynthetic efficiency has not been analyzed previously. The present analysis suggests that overexpression of PDRP would increase photosynthetic efficiency under field conditions.

The sensitivity coefficient of the time constant of stomata opening (*k*_i_) indicated that the speed of stomatal opening was rate limiting from 180 to 600 sec after illumination in maize (Figure 6). This differed from two previous studies indicating that stomatal conductance was not limiting in maize because *C*_i_ was always higher than 100 µmol mol^−1^ during photosynthetic induction (Furbank and Walker, 1985; Usuda and Edwards, 1984). A meta‐analysis of responses of *A* to *C*_i_ across a number of studies in maize indicated that *A* was only CO_2_ saturated at *C*_i_ ≥ 100 µmol mol^−1^ (Pignon and Long, 2020). In the measurements here, *C*_i_ dropped as low as 66 µmol mol^−1^, suggesting that *g*_s_ was a limitation (Figure 4). However, our study used a higher inducing light intensity of 1800 µmol m^−2^ sec^−1^, as compared with 1400 µmol m^−2^ sec^−1^ (Usuda and Edwards, 1984) and 115–1150 µmol m^−2^ sec^−1^ (Furbank and Walker, 1985), and a longer dark treatment time of 30 min, in comparison with 10 and 20 min, respectively. The higher the light intensity used, the lower *C*_i_ appeared during the induction (Furbank and Walker, 1985). The longer dark treatment time used here, was to allow sufficient time for stomata to close and for Rubisco to deactivate.

The present analysis indicated that the activation of Rubisco by Rca is the most important limiting factor after the first few minutes of induction, especially in sorghum with slower Rubisco activation (Figures 5 and 6). In *Oryza sativa* (rice), Rca has been demonstrated to play a crucial role in the regulation of non‐steady‐state photosynthesis (Yamori et al., 2012). Rubisco is arguably the major limiting enzyme of light‐saturated C4 photosynthesis (von Caemmerer, 2000; von Caemmerer et al., 2005; Furbank et al., 1997; Kubien et al., 2003; Wang et al., 2014), and increasing both Rca and Rubisco content have been shown to increase grain yield in maize (Salesse‐Smith et al., 2018; Yin et al., 2014). Hence, based on our simulation and previous studies, increasing the activity of Rubisco and Rca in tandem will increase the photosynthetic efficiency under constant and fluctuating light.

Phosphoenolpyruvate carboxylase (PEPC) appears not to restrict photosynthesis under steady‐state conditions, except under conditions inducing a low *C*_i_, such as drought (Pignon and Long, 2020). However, as *C*_i_ dropped below 100 µmol mol^−1^ during induction (Figure 4b), sensitivity analysis indicated that increasing PEPC would increase photosynthetic efficiency from 180 sec to about 600 sec during induction in maize and sorghum (Figure 7a,c). In sugarcane, however, PEPC limits the steady‐state photosynthetic rate of sugarcane, through its lower *V*_pmax_ compared with maize and sorghum (Figure 7e; Table 2).

### Differences in the limiting factors of photosynthetic induction among species

Furbank et al. (1997) concluded from antisense manipulations that PPDK and Rubisco shared metabolic control of steady‐state light‐saturated photosynthesis in the C4 dicot *Flaveria bidentis*. The limited studies of C4 photosynthesis under fluctuating light have focused on maize. Two early studies indicated that photosynthesis reached a maximum rate after about 15–25 min in maize (Furbank and Walker, 1985; Usuda and Edwards, 1984), which is comparable with the observations and simulations here (Figures 4a and 5).

This study is limited to single accessions of each of three NADP‐ME C4 species. Therefore, it cannot be generalized to the species studied here. However, the examination of individuals from three distinct species of the monophyletic Andropogonae, all C4‐NADPME plants, is likely to have revealed limitations that apply across this key clade of food and energy crops. They therefore point to manipulations that could improve photosynthetic efficiency and yields across the clade. Although there were many similarities, some differences were found. Maize, as perhaps the species most intensively bred for productivity, showed the fastest induction and greatest efficiency of carbon gain over the period of induction, whereas sugarcane was the slowest (Figure 4). Whether these are species characteristics can only be determined by analyzing a wider range of genotypes of each crop. Characterizing within‐species variation would also show the potential for improving non‐steady‐state photosynthesis through breeding. In rice, intraspecific genetic variation in non‐steady‐state photosynthetic efficiency was found to be substantially greater than in the steady state, suggesting an overlooked target for improvement that might similarly be available in these crops (Acevedo‐Siaca et al., 2020a,b).

Maize showed the fastest induction, because of more PDRP and faster τ_Rubisco_ (Table 2), which indicated that maize has faster PPDK and Rubisco activation capacity. However, the stomatal response of maize is slow (Table 2). Here the stomata were one of the major limiting factors during the induction process (Figure 6). This conclusion is different from previous studies (Furbank and Walker, 1985; Usuda and Edwards, 1984), and the possible reasons were explained in the previous section. Speeding up stomatal opening and closing is the key to speed up the photosynthetic response while maintaining water‐use efficiency. New combined thermal and modulated fluorescence techniques now provide a potential high‐throughput means to screen germplasm for this trait (Pignon et al., 2021; Vialet‐Chabrand and Lawson, 2019; Vialet‐Chabrand and Lawson, 2020). Bioengineering for more and smaller stomatal complexes would be another approach (Drake et al., 2013).

For sorghum, the speed of stomatal opening had little effect on *A* during induction (Figure 6). Enzyme activities were the main limiting factors, that is, the concentration of PDRP (i.e. the activation rate of PPDK), the activation rate of Rubisco (τ_Rubisco_) and Rubisco activity (*V*_cmax_) (Figures 6 and 7). Thus, increasing the activity of PDRP, Rca and Rubisco would lead to higher dynamic photosynthesis. However, analysis of water‐use efficiency across a wide range of sorghum germplasm suggests at the species level that the speed of stomatal adjustment is also important (Pignon et al., 2021).

For sugarcane, its dynamic photosynthetic efficiency was co‐limited by many factors, including the rate of stomatal opening, the concentration of PDRP, the activation rate of Rubisco (τ_Rubisco_) and Rubisco activity (*V*_cmax_). In addition, a high control coefficient of PEPC, relative to the other species, was found in sugarcane during induction and in the steady state (Figure 7). Therefore, to improve dynamic photosynthesis, all the limiting factors above may need to be considered. However, with only one accession of each species, our study cannot determine whether these are actual species differences or simply the result of the accessions that were chosen.

### Imbalances in the regulation of C3 and C4 cycles

Coordination between the C3 and C4 cycles is essential to the efficiency of C4 photosynthesis. Leakiness (ϕ) describes the proportion of carbon fixed by PEPC that retrodiffuses back out of bundle‐sheath cells into the mesophyll (Equation 30). It was estimated to be about 0.2 in several C4 species under various environmental conditions (Henderson et al., 1992) and between 0.20 and 0.22 in a recent study of maize (Salesse‐Smith et al. 2018). Our simulated steady‐state ϕ values of the three species lie between 0.2 and 0.3 (Figure 8). In our simulation, the leakiness is predicted to change during the induction through an imbalance in the regulation of the Calvin–Benson cycle and the C4 cycle, especially when the activation of Rubisco is slower (Figure 8, Sorghum). Thus, increasing the activation speed of Rubisco is the first choice to improve efficiency during transitions from dark to light. Although the activation of PPDK is also one of the main limiting factors, increasing the PDRP concentration could not significantly improve photosynthetic efficiency without a larger increase in the speed of Rubisco activation. Overall, this study has identified several potential opportunities for increasing photosynthetic efficiency in these major crops during the frequent light fluctuations that occur in field canopies.

## EXPERIMENTAL PROCEDURES

### Model development

We developed a generic dynamic systems model of C4 photosynthesis from the previous NADP‐ME metabolic model for maize (Wang et al., 2014a,b, Appendix S2). The NADP‐ME metabolic model is an ordinary differential equation model including all individual steps in C4 photosynthetic carbon metabolism. Here, we extended the model to include the post‐translational regulation and temperature response of enzyme activities, together with the dynamics of stomatal conductance and leaf energy balance. The model was implemented in matlab. To save space and make the text more readable, abbreviations have been introduced throughout this section. They can be seen in Table 1.

**Table 1:** Abbreviations

Parameter	Full name	Unit
ϕ	CO_2_ leakiness	Dimensionless
*A*	Net CO_2_ uptake rate	µmol m^−2^ sec^−1^
*C*_i_	Intercellular CO_2_ concentration	μmol mol^–^^1^
*E*	Transpiration rate	mol m^−2^ sec^−1^
*f*_VmRubisco_	The ratio between measured *V*_cmax_ and the maximal Rubisco activities in the model	Unitless
*f*_VmPEPC_	The slope of the linear relationship between measured *V*_pmax_ and the maximal PEPC activities in the model	Unitless
*g*_s_	Stomatal conductance	
*g*_s_Kd_	Rate constant of decreasing stomatal conductance	min^−1^
*g*_s_Ki_	Rate constant of increasing stomatal conductance	min^−1^
*J*_max_	Maximum electron transport capacity	μmol m^−2^ sec^−1^
*K*_o_	Michaelis–Menten constant of Rubisco for O_2_	mbar
*K*_c_	Michaelis–Menten constant of Rubisco for CO_2_	μbar
PAR	Photosynthetically active photon flux	μmol m^−2^ sec^−1^
PEPC	Phosphoenolpyruvate carboxylase	
PDRP	Pyruvate phosphate dikinase regulatory protein	
PPDK	Pyruvate phosphate dikinase	
Rca	Rubisco activase	
*R*_d_	Mitochondria respiration	μmol m^−2^ sec^−1^
τ_Rubisco_	Time constant of Rubisco activation	min
*V*_cmax_	Maximum rubisco activity estimated from measured *A*–*C*_i_ curve	μmol m^−2^ sec^−1^
*V*_pmax_	Maximum PEPC activity estimated from measured *A*–*C*_i_ curve	μmol m^−2^ sec^−1^
*V*_max_	Maximum activity of enzyme	μmol m^−2^ sec^−1^
WUE	Water‐use efficiency (*A*/*E*)	mmol (CO_2_)/mol (H_2_O)

#### Post‐translational regulation of enzyme activity

##### PPDK activation state

The activity of PPDK is regulated by PDRP, which is affected by the level of incident light via ADP concentration (Ashton et al., 1984; Burnell and Hatch, 1985; Chastain, 2010). PDRP is a bifunctional protein kinase/protein phosphatase, catalyzing the reversible phosphorylation of PPDK. The inactivation rate ($V_{\mathrm{PDRP}\_I})$ and activation rate ($V_{\mathrm{PDRP}\_A})$ were calculated by the following equations:

(1) $$V_{\text{PDRP}\_I}=\frac{{\left[\text{PDRP}\right]}_{\text{Mchl}}\cdot k_{\text{cat}\_\text{PDRP}\_I}\cdot {\left[E\right]}_{\text{Mchl}}\cdot {\left[\text{ADP}\right]}_{\text{Mchl}}}{\left({\left[E\right]}_{\text{Mchl}}+K_{m\_\text{PPDK}\_\text{PDRP}\_I}\right)\left({\left[\text{ADP}\right]}_{\text{Mchl}}+K_{m\_\text{ADP}\_\text{PDRP}\_I}\left(1+\frac{{\left[\text{Pyr}\right]}_{\text{Mchl}}}{K_{i\_\text{Pyr}\_\text{PDRP}\_I}}\right)\right)},$$

(2) $$V_{\text{PDRP}\_A}=\frac{{\left[\text{PDRP}\right]}_{\text{Mchl}}\cdot k_{\text{cat}\_\text{PDRP}\_A}\cdot {\left[\text{EP}\right]}_{\text{Mchl}}\cdot {\left[\text{Pi}\right]}_{\text{Mchl}}}{\left({\left[\text{EP}\right]}_{\text{Mchl}}+K_{m\_\text{PPDK}\_\text{PDRP}\_A}\cdot \left(1+\frac{{\left[\text{ADP}\right]}_{\text{Mchl}}}{K_{i\_\text{ADP}\_\text{PDRP}\_A}}\right)\right)\left({\left[\text{Pi}\right]}_{\text{Mchl}}+K_{m\_\text{Pi}\_\text{PDRP}\_A}\right)},$$

where [PDRP]_Mchl_ is the PDRP concentration in the mesophyll cell chloroplasts, and $k_{\mathrm{cat}\_\mathrm{PDRP}\_I}$ and $k_{\mathrm{cat}\_\mathrm{PDRP}\_A}$ are the turnover number of PDRP for the inactivation and activation reaction, respectively. ${\left[E\right]}_{\mathrm{Mchl}}$ is the concentration of active PPDK in the mesophyll chloroplasts; ${\left[\mathrm{EP}\right]}_{\mathrm{Mchl}}$ is the concentration of inactive PPDK in the mesophyll chloroplasts.

Differential equations, parameters and their sources are listed in Appendix S1.

##### Rubisco activation state

The time constant of Rubisco activation was determined from the measured kinetics of photosynthetic gas exchange following transitions from dark to high light, using the method given by Woodrow and Mott (1989) (Equation 27; Figure S4). The differential equation for the transient maximal Rubisco activity is:

(3) $$\frac{dV_{\text{max}\_\text{Rubisco}\_i}}{dt}=\frac{1}{\tau_{\text{Rubisco}}}\left(V_{\text{max}\_\text{Rubisco}\_s}‐V_{\text{max}\_\text{Rubisco}\_i}\right),$$

where τ_Rubisco_ is the rate constant of Rubisco activation catalyzed by Rca. *V*_max_Rubisco_i_ is the transient maximal Rubisco activity; *V*_max_Rubisco_s_ is the steady‐state maximal Rubisco activity, which is related to the Rca concentration, [Rca] (Mott and Woodrow, 2000). The total [Rca] is calculated using measured τ_Rubisco_ (Equation 25; Table 2):

(4) $$\left[\text{Rca}\right]=\frac{k}{\tau_{\text{Rubisco}}},$$

where *k* is a constant, which is 216.9 min mg m^−2^ (Mott and Woodrow, 2000).

**Table 2:** Input parameters for the dynamic C4 photosynthesis model

Parameters	Original value	Reference	Maize	Sorghum	Sugarcane	Method of measurement
Slope_BB_	4.53	Miner et al. (2017)	5.183 ± 0.419	4.843 ± 0.211	4.941 ± 0.177	*A*–*Q* curve
Intercept_BB_	0.020	Miner et al. (2017)	0.036 ± 0.020	0.019 ± 0.013	0.027 ± 0.007	*A*–*Q* curve
*g*_s_ki_ (min^−1^)	0.227	McAusland et al. (2016)	0.127 ± 0.016	0.257 ± 0.063	0.204 ± 0.031	Shade–light dynamics
*g*_s_kd_ (min^−1^)	0.071	McAusland et al. (2016)	0.123 ± 0.026	0.377 ± 0.055	0.221 ± 0.023	Shade–light dynamics
*V*_pmax_ (µmol m^−2^ sec^−1^)	120	Von Caemmerer (2000)	124.128 ± 9.253	133.626 ± 5.678	83.840 ± 4.140	*A*–*C*_i_ curve
*V*_cmax_ (µmol m^−2^ sec^−1^)	60	Von Caemmerer (2000)	49.919 ± 1.847	51.082 ± 2.001	52.783 ± 1.975	*A*–*C*_i_ curve
τ_Rubisco_ (min)	5	Woodrow and Mott (1989)	3.881 ± 1.117	9.714 ± 2.338	4.776 ± 0.316	Dark–light dynamics
*f*_VmPEPC_	1	Assumed	0.72	0.68	0.92	*A*–*C*_i_ curve
*f*_VmRubisco_	0.85	Assumed	0.67	0.56	0.67	*A*–*C*_i_ curve
[PDRP] (µmol)	0.04	Assumed	0.058	0.038	0.037	Dark–light dynamics
Rd (µmol m^−2^ sec^−1^)	1	Von Caemmerer (2000)	2.282	0.979	1.446	Dark–light dynamics

The values were collected from literature or calculated from gas exchange measurements.

Steady‐state maximal Rubisco activity is calculated with the following equations:

(5) $$V_{\text{max}\_\text{Rubisco}\_s}=\frac{V_{\text{max}\_\text{Rubisco}}\cdot {\left[\text{Rca}\right]}_{A}}{K_{\text{activase}}+{\left[\text{Rca}\right]}_{A}},$$

(6) $${\left[\text{Rca}\right]}_{A}=\left[\text{Rca}\right]\cdot a_{\text{Rca}\_s},$$

where $V_{\max\_\mathrm{Rubisco}}$ is the theoretical maximum activity of Rubisco. [Rca]_A_ is the concentration of active Rca, which is regulated by light intensity (Appendix S1). $K_{\mathrm{activase}}$ is a constant, which equals 12.3 mg m^−2^ (Mott and Woodrow, 2000).

##### Activation of enzymes regulated via light intensity

The model used a simplified equation for the light regulation of ATP synthase (ATPase), fructose‐1:6‐bisphosphatase (FBPase), glyceraldehyde‐3‐phosphate dehydrogenase (GAPDH), phosphoribulose kinase (PRK), rubisco activase (Rca) and sedoheptulose‐1:7‐bisphosphatase (SBPase):

(7) $$\frac{dV_{\text{max}\_E\_i}}{dt}=\frac{1}{\tau_{E}}\left(V_{\text{max}\_E\_s}‐V_{\text{max}\_E\_i}\right),$$

(8) $$a_{E\_s}=\text{min}\left(\left(k_{E\_A}\cdot I+c_{E\_A}\right),1\right),$$

(9) $$V_{\text{max}\_E\_s}=V_{\text{max}\_E}\cdot a_{E\_s},$$

where *V*_max_E_i_ is the transient maximal enzyme activity, τ_E_ is the rate constant of the activation of each enzyme and $V_{\max\_E\_s}$ is the steady‐state maximal enzyme activity, as affected by light intensity (I). *k*_E_A_ and *c*_E_A_ are two constants, that is, the slope and intercept of the linear relationship of the proportion of activated enzyme ($a_{E\_s}$) as a function of *I*. $V_{\max\_E}$ is the activity of the enzyme when fully activated.

Although the activation of PEPC is regulated by light via phosphorylation, the whole pathway and parameters of this regulation have not been measured quantitatively. Thus, the dynamics of PEPC activity was as described by Equations (7), (8), (9). Parameters and their sources are listed in Appendix S1.

#### Temperature response of enzymes

In order to simulate the effects of fluctuating leaf temperature with fluctuations in light, the Arrhenius equation (Johnson et al., 1942) and *Q*_10_ function were used to adjust the enzymatic parameters to the actual leaf temperature (*T*_leaf_). The formula used for each parameter was determined based on the availability of experimental data.

The temperature response of the maximum activity of carbonic anhydrase (CA) and PEPC (*V*_max_CA_ and *V*_max_PEPC_) were incorporated into the model using a peaked Arrhenius function (Johnson, Eyring and Williams, 1942):

(10) $$V_{\text{max}\_\text{Enz}1}=V_{\text{max}\_\text{Enz}1\_25}\cdot e^{\frac{E_{a}\cdot \left(T_{\text{leaf}}‐25\right)}{298.15\cdot R\cdot \left(T_{\text{leaf}}+273.15\right)}}\cdot \frac{1+e^{\frac{\left(298.15\cdot \Delta S‐H_{d}\right)}{298.15\cdot R}}}{1+e^{\frac{\left(\left(T_{\text{leaf}}+273.15\right)\cdot \Delta S‐H_{d}\right)}{\left(T_{\text{leaf}}+273.15\right)\cdot R}}},$$

where *E*_a_ is the exponential rate of the rise, *H*_d_ describes the rate of decrease at supraoptimal temperatures and Δ*S* is the entropy factor.

The remperature response of enzymatic parameters of pyruvate phosphate dikinase (*V*_max_PPDK_), electron transport capacity (*J*_max_) and Rubisco (*V*_max_Rubisco_CO2_, *V*_max_Rubisco_O2_/*V*_max_Rubisco_CO2_, *K*_o_ and *K*_c_) were incorporated into the model using an Arrhenius function:

(11) $$V_{\text{max}\_\text{Enz}2}=\;V_{\text{max}\_\text{Enz}2\_25}\cdot e^{\frac{E_{a}\cdot \left(T_{\text{leaf}}‐25\right)}{298.15\cdot R\cdot \left(T_{\text{leaf}}+273.15\right)}},$$

Parameters and sources are listed in Appendix S1.

For other enzymes, a *Q*_10_ function was used to estimate the temperature response of the maximum activity, as described previously (Woodrow and Berry, 1988). Q_10_ was set as 2:

(12) $$V_{\text{max}\_\text{Enz}3}=V_{\text{max}\_\text{Enz}3\_25}\cdot Q_{10\_\text{Enz}}^{\frac{\left(T_{\text{leaf}}‐25\right)}{10}}.$$

#### Dynamic stomatal response

Ball–Berry model parameters for predicting steady‐state stomatal conductance (Ball et al., 1987) were obtained from light response curves measured for each C4 crop evaluated in this study. In the Ball–Berry model, stomatal conductance was with a function of *A*, relative humidity (*RH*) and CO_2_ concentration at the leaf surface (*C*_a_):

(13) $$g_{s\_\text{steady}}={\text{Slope}}_{\text{BB}}\frac{A\cdot RH}{C_{a}}+{\text{Intercept}}_{\text{BB}},$$

where Slope_BB_ is the slope of the relationship between *g*_s_steady_ and *A* × *RH*/*C*_a_ and Intercept_BB_ is the residual stomatal conductance. Slope_BB_ and Intercept_BB_ were estimated by linear regression of $\frac{A\cdot RH}{C_{a}}$ and *g*_s_steady_ from the light response curve (*A*–*Q* curve) measurement.

Dynamic stomatal conductance (*g*_s_) was estimated with the following equation:

(14) $$\frac{dg_{s}}{dt}=k\left(g_{s\_\text{steady}}‐g_{s}\right),$$

where *g*_s_steady_ is the steady‐state stomatal conductance calculated by the Ball–Berry model (Equation 13; Ball et al., 1987); *k* (*k*_i_ or *k*_d_) is the rate constant of the stomata conductance response calculated from the measured stomata dynamics of the three C4 crops, and *k*_i_ and *k*_d_ represent the rate constants of stomata conductance increasing and decreasing, respectively (Table 2; Equation 26).

#### Dynamic leaf energy balance

For leaf energy balance, the equations used in our model were based on the method of Nikolov et al. (1995). According to this model, leaf energy balance takes account of intercepted short‐ and long‐wave radiation, radiative energy loss from the leaf, convection and latent heat loss in transpiration. The net photosynthesis rate (*A*), stomatal conductance and leaf temperature are interdependent. For example, *A* affects stomatal conductance, stomatal conductance affects leaf temperature and leaf temperature affects *A*. Instead of solving these steady‐state circular connections iteratively (Nikolov et al., 1995), a differential equation describes leaf temperature (*T*_leaf_) change (Equation 15):

(15) $$\frac{dT_{\text{leaf}}}{dt}=\frac{PAR_{\text{abs}}+NIR+LR‐\left(H+LE+E+M_{e}\right)}{C_{p}\cdot m_{\text{leaf}}},$$

(16) $$H=2C_{p{\_}_{\text{air}}}g_{\text{bh}}\left(T_{\text{leaf}}‐T_{\text{air}}\right),$$

(17) $$LE=\frac{C_{\text{lv}}g_{l}}{P_{a}}\left(E_{\text{sat}}‐E_{\text{air}}\right),$$

(18) $$E=2\varepsilon \sigma T_{\text{leafK}}^{4},$$

(19) $$M_{e}=0.506A,$$

where ${\mathrm{PAR}}_{\mathrm{abs}}$ is the absorbed photosynthetic active radiation, assuming that 85% of *PAR* is absorbed by the leaf, *NIR* is the absorbed near‐infrared radiation and *LR* is the absorbed long‐wave radiation. Both *NIR* and *LR* were set to zero. *C*_p_ is the specific heat capacity of the leaf, and here we assumed it is the same as the specific heat capacity of water (4.184 J g^−1^ °C^−1^). *m*_leaf_ is the specific leaf fresh weight (g m^−2^) and was set as 198 g m^−2^ for all species based on the measured value of maize leaves (197.9 ± 4.5 g m^−2^). Humidity in the leaf internal air space is assumed to be saturated at the temperature of the leaf. *H* and *LE* are the sensible and latent heat fluxes, respectively. *E* is the emitted long wave radiation and *M*_e_ is the energy consumed in photosynthesis (Nikolov et al., 1995). The boundary layer conductance to heat is calculated as *g*_bh_ = 0.924*g*_b_ (Nikolov et al., 1995). *C*_P_air_ is the specific heat capacity of air (29.3 J mol^−1^ °C^−1^), *C*_lv_ is the latent heat of vaporization of water (44 000 J mol^−1^) and *g*_l_ is the total conductance of the stomata and the boundary layer. ϵ is the leaf emisivity of long‐wave radiation and σ is the Boltzman constant.

#### Boundary layer conductance

Boundary layer conductance was calculated following Nikolov et al. (1995), and both free and forced convection was considered in determining the boundary layer conductance of the leaf. The leaf boundary layer conductance to vapor transport is the maximum of *g*_bf_ and *g*_br_:

(20) $$g_{b}=\text{max}\left(g_{\text{bf}},g_{\text{br}}\right).$$

The forced‐convective and free‐convective leaf boundary layer conductance is computed as:

(21) $$g_{\text{bf}}=c_{f}T_{\text{airk}}^{0.56}{\left[\left(T_{\text{airk}}+120\right)\frac{u}{d_{o}P_{a}}\right]}^{0.5},$$

(22) $$g_{\text{br}}=c_{e}T_{\text{leafk}}^{0.56}{\left(\frac{T_{\text{leafK}}+120}{P_{a}}\right)}^{0.5}{\left(\Delta T\right)}^{0.25},$$

where *d*_o_ is the characteristic dimension of a leaf (leaf width), Δ*T* is the temperature difference between the leaf and the local air (Monteith and Unsworth, 1990), *u* is the wind velocity, and *c*_f_ and *c*_e_ are two constants.

### Gas exchange measurement and parameter estimation

Gas exchange measurements of Maize B73, sugarcane CP88‐1762 and sorghum Tx430 were used to calculate the values of the following photosynthetic parameters: maximum Rubisco activity; maximum PEPC activity; rate constants for stomatal conductance during opening and closing; time constants for Rubisco activation; mitochondrial respiration; concentration of PPDK regulatory protein; and the Ball–Berry slope and intercept (Table 2).

#### Plant material and growth conditions

Maize B73, sugarcane CP88‐1762 and sorghum Tx430 were grown in a controlled‐environment glasshouse at 28°C (day)/24°C (night). Maize and sorghum were grown from seed and sugarcane CP88‐176 was grown from stem cuttings. The positions of the plants in the glasshouse were re‐randomized every week to avoid the influence of environmental variations within the glasshouse. From 25 July to 8 August 2019, six biological replicates were measured in a randomized experimental design for each species in each measurement.

#### Steady‐state gas exchange measurements and parameter estimation

Leaf gas exchange of the youngest fully expanded leaf was measured on plants at 30–35 days old with a gas exchange system (LI‐6800; LI‐COR, Lincoln, NE, USA). The leaf chamber temperature was set at 28°C, with a water vapor pressure deficit of 1.32 KPa and a flow rate of 500 µmol sec^−1^ for all gas exchange measurements.

For the response of *A* to intracellular CO_2_ concentration curves (*A*–*C*_i_ curves), the leaf was acclimated to a light intensity of 1800 µmol m^−2^ sec^−1^ and a CO_2_ concentration of 400 µmol mol^−1^. After both *A* and *g*_s_ reached steady state, the CO_2_ concentration of the influent gas was varied in the following sequence: 400, 300, 200, 120, 70, 40, 20, 10, 400, 400, 400, 600, 800, 1200 and 1500 µmol mol^−1^.

The maximum Rubisco activity (*V*_cmax_) and maximum PEPC activity (*V*_pmax_) were estimated from the *A*–*C*_i_ curves using the equations of von Caemmerer (2000). In order to obtain the relationship between estimated *V*_pmax_ and the theoretical maximal PEPC activity (*V*_max_PEPC_) in our model, and similarly the relationship between *V*_cmax_ and the theoretical maximal Rubisco activity (*V*_max_Rubisco_), we introduced two variables (*f*_vpmax_ and *f*_vcmax_) into the simulation:

(23) $$V_{\text{max}\_\text{PEPC}}=\frac{1}{f_{\text{vpmax}}}V_{\text{pmax}},$$

(24) $$V_{\text{max}\_\text{Rubisco}}=\frac{1}{f_{\text{vcmax}}}V_{\text{cmax}},$$

Both *f*_vpmax_ and *f*_vcmax_ were estimated by minimizing the sum ($S_{\mathrm{fvPEPC}}\;\mathrm{and}\;S_{\mathrm{fvcmax}}$) of the squared differences between the estimated *A* (*A*_e_ *__C_*_i_) from the dynamic model and the measured *A* (*A*_m_ *__C_*_i_), in response to intercellular CO_2_ (*A–C*_i_ curve), using the least‐squares method for each species:

(25) $$S_{\text{fvPEPC}}={\left(s_{\text{Ae}\_\text{Ci}}\left(f_{\text{vPEPC}}\right)‐s_{\text{Am}\_\text{Ci}}\right)}^{2},$$

(26) $$S_{\text{fvcmax}=}\sum {\left(A_{e\_\text{Ci}}\left(f_{\text{vcmax}}\right)‐A_{m\_\text{Ci}}\right)}^{2}.$$

*f*_vPEPC_ was estimated using the initial slope ($s_{\mathrm{Am}\_\mathrm{Ci}}$) of the measured *A*–*C*_i_ curve (CO_2_ air = 120, 70, 40, 20, 10 µmol mol^−1^); *f*_vcmax_ was estimated using CO_2_‐saturated *A*_m_Ci_ (CO_2_ air = 800, 1200 and 1500 µmol mol^−1^) (Figure S3). The steady‐state *V*_max_ of the other enzymes of C4 and C3 metabolism in Figure 1 were scaled for each species with *f*_vpmax_ and *f*_vcmax_, respectively.

To define the response of *A* to light intensity (*A*–*Q* curves), the leaf was acclimated to a light intensity of 1800 µmol m^−2^ sec^−1^ and a CO_2_ concentration of 400 µmol mol^−1^. After leaf gas exchange reached the steady state, the light intensity in the chamber was changed in the following sequence: 2000, 1500, 1000, 500, 300, 200, 100 and 50 µmol m^−2^ sec^−1^. The gas exchange data were logged after 5 min to ensure that there was enough time for the transpiration, and therefore stomatal condcutance, to reach the steady state at each light level. Ball–Berry model parameters (Ball et al., 1987) were estimated by the linear regression of $\frac{A\cdot RH}{C_{a}}$ and *g*_s_steady_ from data from the *A*–*Q* curves, including the prediction of steady‐state stomatal conductance (*g*_s_steady_) for each species (Equation 13).

#### Dynamic gas exchange measurements and parameter estimation

Gas exchange during photosynthetic induction was measured in the transition from darkness to high light (1800 μmol m^−2^ sec^−1^) to determine the kinetics of Rubisco activation in these C4 crops ($\tau_{\mathrm{Rubisco}}$). The leaf was first acclimated to darkness for 30 min, with a CO_2_ concentration of 400 µmol mol^−1^ and then the light intensity was changed to 1800 μmol m^−2^ sec^−1^ for 30 min, which was more than sufficient time for leaf CO_2_ uptake and stomatal conductance to reach the steady state. Leaf gas exchange was logged before the light was turned on, and then logged every 10 sec for the following 30 min. The time constant of Rubisco activation (τ_Rubisco_) was estimated from the linear portion of the semi‐logarithmic plot of photosynthesis with time (Woodrow and Mott, 1989, 1993; Figure S4). The slope of this portion was determined by the linear regression of the data between 3 and 7 min. The value of $\tau_{\mathrm{Rubisco}}$was calculated as:

(27) $$\tau_{\text{Rubisco}}=‐\frac{1}{\text{slope}}.$$

The calculated values of the three C4 species are listed in Table 2.

To further evaluate the response of gas exchange in C4 plants under fluctuating light, following this 30 min of induction the responses to the transition from high to low and back to high light (i.e. relaxation curves followed by induction curves) were measured. This involved decreasing light to 200 µmol m^−2^ sec^−1^ PPFD for 30 min and then returning to 1800 µmol m^−2^ sec^−1^ PPFD for an additional 30 min. Gas exchange was recorded every 10 sec.

Rate constants were calculated for *g*_s_ increase on transfer from low light (200 µmol m^−2^ sec^−1^ PPFD) to high light (*k*_i_), and again for the decrease in *g*_s_ on return to low light (*k*_d_). The measured time series for stomatal conductance changes were fitted with the following equation (Vialet‐Chabrand et al., 2017):

(28) $$g_{s}=\left(g_{\text{max}}‐g_{0}\right)e^{‐kt}+g_{0},$$

where *g*_max_ is the maximum stomata conductance, *g*_0_ is the minimum stomata conductance, *t* is time and *k* (*k*_i_ or *k*_d_) is the rate constant of *g*_s_. *g*_max_, *g*_0_ and *k* were estimated using Equation 28 by the curve fitting function (fit) in matlab (MathWorks, https://www.mathworks.com).

Mitochondrial respiration (*R*_d_) was estimated from the measured CO_2_ efflux after 30 min of dark adaptation. The concentration of PDRP was estimated by minimizing the difference between the estimated *A* (*A*_e_t_) from the dynamic model and the measured *A* (A_m_t_) at the beginning of the induction using the least‐squares method, which minimizes the sum ($S_{\mathrm{PDRP}}$) of the squared difference between estimated and measured *A* in the beginning of the photosynthetic induction (Figure S5). Data points from 1–3 min of the induction were used.

(29) $$S_{\text{PDRP}}=\sum {\left(A_{e\_t}\left(\left[\text{PDRP}\right]\right)‐A_{m\_t}\right)}^{2}$$

#### Model parameterization

The model took the following 11 photosynthetic parameters estimated from measured gas exchange data as input variables: maximum Rubisco activity (*V*_cmax_ and *f*_vcmax_); maximum PEPC activity (*V*_pmax_ and *f*_vpmax_); the rate constant of stomata conductance increase and decrease (*k*_i_ and *k*_d_); the time constant of Rubisco activation (τ_Rubisco_); mitochondrial respiration (*R*_d_); [PDRP]; and the Ball–Berry slope (Slope_BB_) and intercept (Intercept_BB_) (Table 2). The estimation methods of the input variables were described in the previous section (Gas exchange measurement and parameter estimation). Parameters and equations affected by the input variables are listed in Appendix S1.

### Model prediction

#### CO_2_ uptake rate (*A*) and leakiness (ϕ) calculation

During the simulation, metabolite concentrations and reaction rates were extracted from the model. The velocity of CO_2_ flowing into the leaf via stomata was used to represent *A*. Leakiness (ϕ) describes the proportion of carbon fixed by PEPC that subsequently leaks out of the bundle sheath cells. Thus, ϕ was calculated as:

(30) $$\phi =\frac{v_{{\text{CO}}_{2}\_\text{leak}}}{v_{\text{PEPC}}}=\frac{P_{{\text{CO}}_{2}\_\text{pd}}\left({\left[{\text{CO}}_{2}\right]}_{\text{BSC}}‐{\left[{\text{CO}}_{2}\right]}_{\text{MC}}\right)}{v_{\text{PEPC}}},$$

where the CO_2_ leak rate (*v*_CO2_leak_) is determined by the permeability of CO_2_ through plasmodesmata (*P*_CO2_pd_) and the concentration gradient of CO_2_ between bundle sheath cytosol and mesophyll cytosol, [CO_2_]_BSC_–[CO_2_]_MC_, and *v*_PEPC_ is the velocity of carbon fixation by PEPC.

#### Sensitivity analysis

The sensitivity coefficient (*SC*_p_) gives the relative fractional change in the simulated result with a fractional change in the input variable (*p*), *SC*_p_ is the partial derivative used to describe how the output estimate varies with changes in the values of the input parameter (*p*), where the output in this study is the estimated leaf CO_2_ uptake rate (*A*):

(31) $$SC_{p}=\frac{\partial A}{\partial p}\frac{p}{A}\;\approx \;\frac{A^{+}‐A^{‐}}{0.02\cdot A},$$

where the variable (*p*) was both increased and decreased by 1% individually in the model to calculate the new value of *A* (*A* ^+^ and *A* ^−^, respectively) in order to identify the parameters influencing *A*.

The flux control coefficient of each enzyme (*FCC*) was also estimated by Equation 31, using the maximal activity (*V*_max_E_) of the enzyme as the variable (*p*).

## AUTHOR CONTRIBUTIONS

SPL and YW designed the study. YW performed the computational analysis. YW conducted the gas exchange measurements. YW and KC implemented the literature data collection. SPL, YW and KC wrote the article.

## CONFLICT OF INTEREST

The authors declare that they have no conflicts of interest associated with this work.

## Supporting information


**Figure S1**. Simulated photosynthetic induction using metabolic model without post‐translational regulation of enzymes and delay of stomata conductance.
**Figure S2**. Estimated influence of mutase and enolase on photosynthetic induction.
**Figure S3**. Estimation of *f*_vPEPC_ and *f*_vRubisco_ using least‐squares method.
**Figure S4**. Semilogarithmic plot of the difference between photosynthesis (*A*) and maximum photosynthesis (*A*_f_) as a function of time.
**Figure S5**. Estimation of PPDK regulatory protein concentration, [PDRP], using measured photosynthetic induction curves.
**Figure S6**. Measured CO_2_ response curves and light response curves of maize B73, sorghum Tx430 and sugarcane CP88‐1762.
**Figure S7**. Calculated Ball–Berry slope and intercept using gas exchange data from the light response curves of maize, sorghum and sugarcane.

**Appendix S1**. New modules of the dynamic photosynthesis model.

**Appendix S2**. Equations and parameters of the C4 metabolic model (Wang et al., 2014).

## Data Availability

All measurements made here are freely available from the Illinois DataBank at https://doi.org/10.13012/B2IDB‐2694900_V1. All scripts for the model and associated files are available at https://github.com/yuwangcn/C4_dynamic_model.
