# Supplementary material for: Towards a dynamic photosynthesis model to guide yield improvement in C4 crops
Source: Plant J. 2021 Aug 6;107(2):343–59. doi: 10.1111/tpj.15365 (PMC9291162; doi:10.1111/tpj.15365)
Supplement: Supplementary file 1 — Figure S1. Simulated photosynthetic induction using metabolic model without post‐translational regulation of enzymes and delay of stomata conductance. Figure S2. Estimated influence of mutase and enolase on photosynthetic induction. Figure S3. Estimation of f vPEPC and f vRubisco using least‐squares method. Figure S4. Semilogarithmic plot of the difference between photosynthesis (A) and maximum photosynthesis (A f) as a function of time. Figure S5. Estimation of PPDK regulatory protein concentration, [PDRP], using measured photosynthetic induction curves. Figure S6. Measured CO2 response curves and light response curves of maize B73, sorghum Tx430 and sugarcane CP88‐1762. Figure S7. Calculated Ball–Berry slope and intercept using gas exchange data from the light response curves of maize, sorghum and sugarcane. [file TPJ-107-343-s001.docx]

**Supplemental figures**





Figure S1 Simulated photosynthetic induction using metabolic model without posttranslational regulation of enzymes and delay of stomata conductance. a) Net photosynthesis rate; b) leakiness; c) relative concentrations of C4 cycle metabolites; d) relative concentration of Calvin-Benson cycle metabolites.





Figure S2 Estimated influence of mutase and enolase on photosynthetic induction using metabolic model without posttranslational regulation of enzymes and delay of stomata conductance.

Figure S3 Estimation of *f_vPEPC_* and *f_vRubisco_* using least squares method. The slope of measured A-Ci curve was used to estimate *f_vPEPC_* and the plateau of A-Ci curve was used for *f_vRubisco ._*

Figure S4 Semilogarithmic plot of the difference between the photosynthesis (A) and maximum photosynthesis (A_f_) as a function of time. Time courses for photosynthesis were measured following a change in PPFD from 0 to 1800 µmol m^-2^ s^-1^. Data between 3-7 min of the measured curves was used to estimate the τ_Rubisco_ (Table 2)

Figure S5 Estimation of PPDK regulatory protein concentration *([PDRP])* using measured photosynthetic induction curves. PDRP concentration was estimated using least squares method, minimized the sum of square of the difference between dynamic model estimated and measured CO_2_ uptake rate in the beginning of the photosynthetic induction (1 - 3 min).

.





Figure S6 Measured CO_2_ response curves and light response curves of maize B73, sorghum Tx430 and sugarcane CP88-1762. Error bars represent standard errors, six replicates were measured for each species.


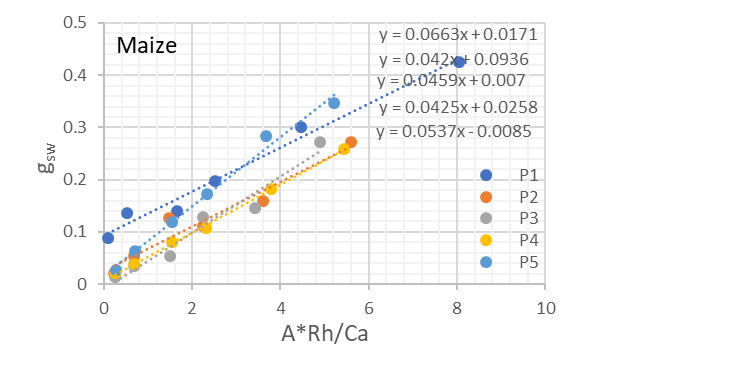

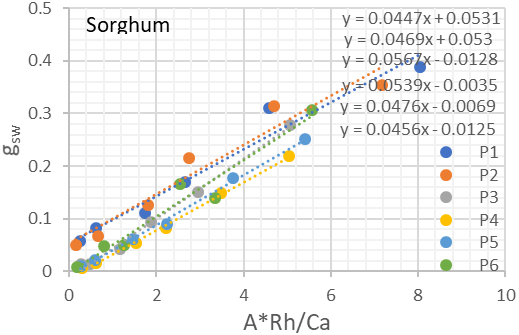

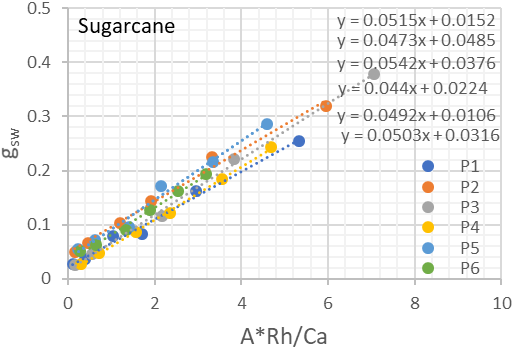


Figure S7 Calculated Ball-Berry slope and intercept using gas exchange data from light response curves of maize, sorghum and sugarcane. Color represents each individual measurement.
