# Supplementary material for: Towards a dynamic photosynthesis model to guide yield improvement in C4 crops
Source: Plant J. 2021 Aug 6;107(2):343–59. doi: 10.1111/tpj.15365 (PMC9291162; doi:10.1111/tpj.15365)
Supplement: Supplementary file 2 — Appendix S1. New modules of the dynamic photosynthesis model. [file TPJ-107-343-s002.docx]

**Appendix File 1 New modules of the dynamic photosynthesis model**

Contents

[1. Post-translational regulation of enzyme activity 1](#_Toc54883190)

[1.1. PPDK activity regulation 1](#_Toc54883191)

[1.2. Rubisco activity regulation 3](#_Toc54883192)

[1.3. Activation of enzymes regulated via thioredoxin redox state 4](#_Toc54883193)

[2. Temperature response of enzymes 5](#_Toc54883194)

[3. Leaf physiology 7](#_Toc54883195)

[3.1. Dynamic stomatal response 7](#_Toc54883196)

[3.2. Boundary layer conductance 7](#_Toc54883197)

[3.3. Dynamic Leaf energy balance 8](#_Toc54883198)

[4. Model parameterization 9](#_Toc54883199)

[Reference 13](#_Toc54883200)

# Post-translational regulation of enzyme activity

## PPDK activity regulation

The pyruvate phosphate dikinase (PPDK) activity is regulated by the PPDK regulatory protein (PDRP) which is affected by the level of incident light via ADP concentration (Ashton *et al.*, 1984, Burnell and Hatch, 1985, Chastain, 2010). PDRP is a bifunctional protein kinase/protein phosphatase, catalyzing reversible phosphorylation of PPDK. The inactivation rate( $V_{PDRP\_I})$and activation rate ($V_{PDRP\_A})$were calculated by the following equations:

$V_{PDRP\_I}=\frac{{[PDRP]}_{Mchl}\cdot k_{cat\_PDRP\_I}\cdot\left[ E \right]_{Mchl}\cdot\left[ ADP \right]_{Mchl}}{(\left[ E \right]_{Mchl}+K_{m\_PPDK\_PDRP\_I})(\left[ ADP \right]_{Mchl}+K_{m\_ADP\_PDRP\_I}(1+\frac{\left[ Pyr \right]_{Mchl}}{K_{i\_Pyr\_PDRP\_I}}))}$ (1)

$V_{PDRP\_A}=\frac{{[PDRP]}_{Mchl}\cdot k_{cat\_PDRP\_A}\cdot\left[ EP \right]_{Mchl}\cdot\left[ Pi \right]_{Mchl}}{(\left[ EP \right]_{Mchl}+K_{m\_PPDK\_PDRP\_A}\cdot(1+\frac{\left[ ADP \right]_{Mchl}}{K_{i\_ADP\_PDRP\_A}}))(\left[ Pi \right]_{Mchl}+K_{m\_Pi\_PDRP\_A})}$ (2)

Where *[PDRP]_Mchl_* is the PDRP concentration in mesophyll cell chloroplast, $k_{cat\_PDRP\_I}$ and $k_{cat\_PDRP\_A}$ are the turnover number of PDRP for the inactivation and activation reaction respectively. $\left[ E \right]_{Mchl}$is the concentration of active PPDK in mesophyll chloroplast; $\left[ EP \right]_{Mchl}$ is the concentration of inactive PPDK in mesophyll chloroplast.

The differential equations for active PPDK and inactive PPDK are:

$\frac{d\left[ E \right]_{Mchl}}{dt}={\frac{1}{{Vol}_{Mchl}}(V}_{PDRP\_A}-V_{PDRP\_I})$ (3)

$\frac{d\left[ EP \right]_{Mchl}}{dt}=\frac{1}{{Vol}_{Mchl}}(V_{PDRP\_I}-V_{PDRP\_A})$ (4)

*V_olMchl_* is the volume of mesophyll cell chloroplast per unit leaf area (square meter).

The total PPDK concentration in mesophyll chloroplast( $\left[ PPDK \right]_{Mchl})$ is calculated by following equation:

$\left[ PPDK \right]_{Mchl}=\frac{V_{max\_PPDK}}{K_{cat\_PPDK}\cdot{Vol}_{Mchl}}$ (5)

*Where V_max_PPDK_* is the theoretical maximum activity of PPDK*; K_cat_PPDK_ i*s the turnover number of PPDK.

The transient maximum activity of PPDK ($V_{max\_PPDK\_i}$) is:

$V_{max\_PPDK\_i}=V_{max\_PPDK}\frac{\left[ E \right]_{Mchl}}{\left[ E \right]_{Mchl}+\left[ EP \right]_{Mchl}}$ (6)

Parameters and their sources are listed in Table S1

**Table S1 Parameters of PPDK activation regulated by PDRP**

| Parameter | Value | Reference |
| --- | --- | --- |
| Inactivation |  |  |
| *K_m_ADP_PDRP_I_* | 0.052mM | (Burnell and Hatch, 1985, Roeske and Chollet, 1987) |
| *K_m_PPDK_PDRP_I_* | 0.0012mM | (Burnell and Hatch, 1985) |
| *K_i_Pyr_PDRP_I_* | 0.08mM | (Burnell and Hatch, 1985) |
| Activation |  |  |
| *K_m_Pi_PDRP_A_* | 0.65mM | (Burnell and Hatch, 1985, Roeske and Chollet, 1987) |
| *K_m_PPDK_PDRP_A_* | 0.0007mM | (Burnell and Hatch, 1985) |
| *K_i_ADP_PDRP_A_* | 0.085 | (Burnell and Hatch, 1985) |
| *K_cat_PDRP_I_* | 1.125 s-1 | (Roeske and Chollet, 1987) |
| *K_cat_PDRP_A_* | 0.578 s-1 | (Roeske and Chollet, 1987) |
| Molecular weight of PDRP | 45 kDa | (Chastain *et al.*, 2018) |
| V_max_PPDK_ | Eq. 16 and Table 5 |  |
| K_cat_PPDK_ | Specific activity*MW |  |
| Specific activity of PPDK | 4.1 µmol/min/mg | (Chastain *et al.*, 2000) |
| MW of PPDK | 95000 Da | (Chastain *et al.*, 2000) |
| Vol_Mchl_ | 0.02 L m^-2^ | (Wang *et al.*, 2014) |

## Rubisco activity regulation

The time constant of Rubisco activation was determined from the kinetics of photosynthesis following transitions from dark to high light (Woodrow and Mott, 1989), the differential equations of Rubisco activity is:

$\frac{dV_{max\_Rubisco\_i}}{dt}=\frac{1}{\tau_{Rubisco}}(V_{max\_Rubisco\_s}-V_{max\_Rubisco\_i})$ (7)

Where *τ_Rubioco_* is the rate constant of Rubisco activation catalyzed by Rubisco activase. *V_max_Rubisco_i_* is the transient maximal Rubisco activity; *V_max_Rubisco_s_* is the steady-state maximal Rubisco activity which is related to the Rubisco activase concentration ([*Rac*]) (Mott and Woodrow, 2000). The total Rubisco activase concentration ([*Rac*]) is calculated using measured *τ_Rubioco_*

$[Rca]=\frac{k}{\tau_{Rubisco}}$ (8)

Steady-state maximal Rubisco activity is calculated with the following equations**.**

$V_{max\_Rubisco\_s}=\frac{V_{max\_Rubisco}{[Rca]}_{A}}{K_{activase}+{[Rca]}_{A}}+c$ (9)

${[Rca]}_{A}=[Rca]*a_{Rca\_s}$ (10)where $V_{max\_Rubisco}$ is the theoretical maximum activity of Rubisco. [Rca]_A_ is the concentration of active rubisco activase, which is regulated by thioredoxin redox state (Section 1.3).

$K_{activase} and$k are two constants, which equal 12.3 mg m^-2^ and 216.9 min mg m^-2^, respectively (Mott and Woodrow, 2000).

## Activation of enzymes regulated via light intensity

The model used a simplified equation for light regulation of ATP synthase (ATPase), sedoheptulose-1:7-bisphosphatase (SBPase), fructose-1:6-bisphosphatase (FBPase), phosphoribulose kinase (PRK) and glyceraldehyde-3-phosphate dehydrogenase (GAPDH),and Rubisco activase (Rca):

$\frac{dV_{max\_E\_i}}{dt}=\frac{1}{\tau_{E}}(V_{max\_E\_s}-V_{max\_E\_i})$ (11)

$a_{E\_s}=min \{(k_{E_{A}}\cdot I+c_{E_{A}}), 1\}$ (12)

$V_{max\_E\_s}=V_{max\_E}\cdot a_{E\_s}$ (13)

Where *V_max_E_i_* is the transient maximal enzyme activity, τ_E_ is the rate constant of the activation of each enzyme, $V_{m\_E\_s}$ is the steady-state maximal enzyme activity, as affected by light intensity (*I*). *k_E_A_* and *c_E_A_* are two constants, i.e. the slope and intercept of the linear relationship. $V_{max\_E}$ is the theoretical maximum enzyme activity. $a_{E\_s}$ is the proportion of activated enzyme.

Although activation of PEPC is regulated by light via phosphorylation, the whole pathway and parameters of this regulation haven’t been quantitatively measured. Thus, the dynamics of PEPC activity could was described by Eq. 11-13.

**Table S2 Parameters of enzyme activation regulated by light intensity**

| Parameter | Value | Reference |
| --- | --- | --- |
| *τ_Rubioco_* | variable | measured |
| *τ_RCA_* | 0.7594 | (Yoshida and Hisabori, 2018) |
| *τ_FBPase_* | 1.878 min | (Yoshida and Hisabori, 2018) |
| *τ_SBPase_* | 3.963 min | (Yoshida and Hisabori, 2018) |
| *τ_ATPsynthase_* | 0.5 min | Assumed |
| *τ_GAPDH_* | 1.0 min | Assumed |
| *τ_PRK_* | 1.0 min | Assumed |
| *τ_NADPMDH_* | 0.965 min | (Usuda *et al.*, 1984) |
| *τ_PEPC_* | 2.0 min | Assumed |
| *k_RCA_* | 0.0017 | Assumed the same as *k_FBPase_* |
| *k_FBPase_* | 0.0017 | (Usuda *et al.*, 1984) |
| *k_SBPase_* | 0.0017 | Assumed the same as *k_FBPase_* |
| *k_ATPsynthase_* | 0.0017 | Assumed the same as *k_FBPase_* |
| *k_GAPDH_* | 0.0017 | Assumed the same as *k_FBPase_* |
| *k_PRK_* | 0.0017 | Assumed the same as *k_FBPase_* |
| *k_NADPMDH_* | 0.0017 | (Usuda *et al.*, 1984) |
| *k_PEPC_* | 0.0017 | Assumed the same as *k_NADPMDH_* |
| *c_RCA_* | 0.06 | Assumed |
| *c_FBPase_* | 0.05 | Assumed the same as c*_NADPMDH_* |
| *c_SBPase_* | 0.05 | Assumed the same as c*_NADPMDH_* |
| *c_ATPsynthase_* | 0.05 | Assumed the same as c*_NADPMDH_* |
| *c_GAPDH_* | 0.05 | Assumed the same as c*_NADPMDH_* |
| *c_PRK_* | 0.05 | Assumed the same as c*_NADPMDH_* |
| *c_NADPMDH_* | 0.05 | (Usuda *et al.*, 1984) |
| *c_PEPC_* | 0.05 | Assumed the same as c*_NADPMDH_* |
|  |  |  |

# Temperature response of enzymes

To simulate the effect of temperature, the Arrhenius equation and Q_10_ function were used to adjust the enzymatic parameters to the actual leaf temperature (T_leaf_). For each parameter, which formula to use was determined based on available experimental data from literatures.

Temperature response of the maximum activity of carbonic anhydrase (CA) and PEP carboxylase (PEPC) (*V_m_CA_* and *V_m_PEPC_*) were incorporated into the model using a peaked Arrhenius Function (Johnson, Eyring & Williams 1942).

$V_{max\_CA}=V_{max\_CA\_25}\cdot e^{\frac{E_{a\_Vm\_CA}\cdot(T_{leaf}-25)}{298.15\cdot R\cdot(T_{leaf}+273.15)}}\cdot\frac{1+e^{\frac{(298.15\cdot{\Delta S}_{Vm\_CA}-H_{d\_Vm\_CA})}{298.15\cdot R}}}{1+e^{\frac{((T_{leaf}+273.15)\cdot{\Delta S}_{Vm\_CA}-H_{d\_Vm\_CA})}{(T_{leaf}+273.15)\cdot R}}}$ (14)

$V_{max\_PEPC}=V_{max\_PEPC\_25}\cdot e^{\frac{E_{a\_Vm\_PEPC}\cdot(T_{leaf}-25)}{298.15\cdot R\cdot(T_{leaf}+273.15)}}\cdot\frac{1+e^{\frac{(298.15\cdot{\Delta S}_{Vm\_PEPC}-H_{d\_Vm\_PEPC})}{298.15\cdot R}}}{1+e^{\frac{((T_{leaf}+273.15)\cdot{\Delta S}_{Vm\_PEPC}-H_{d\_Vm\_PEPC})}{(T_{leaf}+273.15)\cdot R}}}$ (15)

Where *E_a_* is the exponential rate of rise; *H*_d_ describes the rate of decrease of the function above ‘the optimum; *ΔS* is the entropy factor

Temperature response of enzymatic parameters of pyruvate phosphate dikinase (*V_max_PPDK_* ), electron transport capacity (*J_max_*) and Rubisco (*V_max_Rubisco_CO2_*, *V_max_Rubisco_O2_/V_max_Rubisco_CO2_*, *K_o_* and *K_c_*) were incorporated into the model using an Arrhenius Function.

$V_{max\_PPDK}=\text{ V}_{max\_PPDK\_25}\cdot e^{\frac{E_{a\_PPDK}\cdot\left( T_{leaf}-25 \right)}{298.15\cdot R\cdot(T_{leaf}+273.15)}}$ (16)

$V_{max\_Rubisco}=\text{ V}_{max\_Rubisco\_25}\cdot e^{\frac{E_{a\_Rubisco}\cdot\left( T_{leaf}-25 \right)}{298.15\cdot R\cdot(T_{leaf}+273.15)}}$ (17)

$V_{m\_o/c}=\text{ V}_{m\_o/c\_25}\cdot e^{\frac{E_{a\_Vm\_o/c}\cdot\left( T_{leaf}-25 \right)}{298.15\cdot R\cdot(T_{leaf}+273.15)}}$ (18)

$K_{o}=\text{K}_{o\_25}\cdot e^{\frac{E_{a\_Ko}\cdot\left( T_{leaf}-25 \right)}{298.15\cdot R\cdot(T_{leaf}+273.15)}}$ (19)

$K_{c}=\text{K}_{c\_25}\cdot e^{\frac{E_{a\_Kc}\cdot\left( T_{leaf}-25 \right)}{298.15\cdot R\cdot(T_{leaf}+273.15)}}$ (20)

$J_{max}=\text{ J}_{max\_25}\cdot e^{\frac{E_{a\_Jmax}\cdot\left( T_{leaf}-25 \right)}{298.15\cdot R\cdot(T_{leaf}+273.15)}}$ (21)

For other enzymes, a *Q_10_* function was used to estimate the temperature response of the maximum activity, as described previously (Woodrow and Berry, 1988). Q_10_ was set as 2.

$V_{max\_Enz3}=V_{max\_Enz3\_25}\cdot Q_{10\_Enz}^{\frac{(T_{leaf}-25)}{10}}$ (22)

Parameters and sources are listed in the following table (Table S3)

**Table 3 Temperature response parameters of photosynthetic enzymes**

| Reaction | Parameter | Value | Reference |
| --- | --- | --- | --- |
| CA | Ea_V_m_CA_ | 40.9 kJ mol^-1^ | (Boyd *et al.*, 2015) *S. viridis* |
|  | dS_V_m_CA_ | 0.21 kJ mol^-1^K^-1^ | (Boyd *et al.*, 2015) *S. viridis* |
|  | Hd_V_m_CA_ | 64.5 kJ mol^-1^ | (Boyd *et al.*, 2015) *S. viridis* |
| PEPC | Ea_V_m_PEPC_ | 94.8 kJ mol^-1^ | (Boyd *et al.*, 2015) *S. viridis* |
|  | dS_V_m_PEPC_ | 0.25 kJ mol^-1^K^-1^ | (Boyd *et al.*, 2015) *S. viridis* |
|  | Hd_V_m_PEPC_ | 73.3 kJ mol^-1^ | (Boyd *et al.*, 2015) *S. viridis* |
| PPDK | Ea_V_m_PPDK_ | 58.1kJ mol^-1^ | (Wang *et al.*, 2008) *Maize* |
| Rubico | Ea_V_m_Rubisco_ | 78 kJ mol^-1^ | (Boyd *et al.*, 2015) *S. viridis* |
|  | Ea_Kc | 64.2 kJ mol^-1^ | (Boyd *et al.*, 2015) *S. viridis* |
|  | Ea_Ko | 10.5kJ mol^-1^ | (Boyd *et al.*, 2015) *S. viridis* |
|  | V_m_O/C_ _25 | 0.18 | (Boyd *et al.*, 2015) *S. viridis* |
|  | Ea_ V_m_O/C_ | 55.3 kJ mol^-1^ | (Boyd *et al.*, 2015) *S. viridis* |
| Jmax | Ea_J_max_ | 43.1 kJ mol^-1^ | (Bernacchi *et al.*, 2003) *N. tobaccum* |
| Others | Q_10_E_ | 2 | Assumed |

R=8.3144598 (m^2^ kg s^-2^ K^-1^ mol^-1^)

# Leaf physiology

## Dynamic stomatal response

Dynamic stomatal conductance (*g_s_*) was estimated by the following equation:

$\frac{dg_{s}}{dt}=k(g_{s\_steady}-g_{s})$ (23)

where *g_s_steady_* is the steady-state stomata conductance calculated by the Ball-Berry model (Eq. 24) (Ball *et al.*, 1987); *k* ( *k_i_* or *k_d_* ) is the rate constant of stomata conductance response calculated from measured stomata dynamics of the three C4 crops, *k_i_* and *k_d_* represent the rate constant of stomata conductance increasing and decreasing, respectively (Table 2). Ball-Berry model parameters for predicting steady-state stomatal conductance (Ball *et al.*, 1987) were obtained from light response curves measured for each C4 crop evaluated in this study. In the Ball-Berry model, stomatal conductance is correlated with A, relative humidity (RH) and CO_2_ concentration at the leaf surface (*C_a_*):

$g_{s\_steady}={Slope}_{BB}\frac{A\cdot RH}{C_{a}}+{Intercept}_{BB}$ (24)

Where *Slope_BB_* is the slope of the relationship between *g_s_steady_* and *A*RHs/Ca*. *Intercept_BB_* is the residual stomatal conductance. *Slope_BB_* and *Intercept_BB_* were estimated by linear regression.

## Boundary layer conductance

Boundary layer conductance was calculated following Nikolov et al. (1995), both free and forced convection was considered in determining the boundary layer conductance of leaf. The leaf boundary layer conductance to vapor transport is the maximum of g_bf_ and g_br_

$g_{bw}=max(g_{bf},g_{br})$ (25)

Forced-convective and free-convective leaf boundary layer conductance is computed as

$g_{bf}=c_{f}T_{airk}^{0.56}{[(T_{airk}+120)\frac{u}{d_{o}P_{a}}]}^{0.5}$ (26)

$g_{br}=c_{e}T_{leafk}^{0.56}{(\frac{T_{leafK}+120}{P_{a}})}^{0.5}{(\Delta T)}^{0.25}$ (27)

where *d_o_* is the characteristic dimension of a leaf (leaf width). ΔT is the virtual temperature difference between leaf and the local air (Monteith and Unsworth, 1990). u is the wind velocity; *c_f_* and *c_e_* are two constants.

The CO_2_ conductance from air to intercellular space $g_{c\_t}$ is calculated as

$g_{c\_t}=\frac{{g_{b}+g}_{s}}{g_{b}\cdot g_{s}}$ (28)

$g_{b}=\frac{g_{bw}}{1.37}$ (29)

Where *g_b_* is the leaf boundary layer conductance to CO_2._

Then the differential equation for intercellular CO_2_ concentration changes is:

$\frac{dC_{i}}{dt}=(v_{c\_in}-v_{inf})\frac{1}{{Vol}_{i}}$ (30)

${v_{c\_in}=g}_{c\_t}(C_{a}-C_{i})$ (31)

Where $v_{c\_in}$*is* the rate of CO_2_ diffusion from atmosphere to intercellular space*.* $v_{inf}$: The rate CO_2_ diffuse from intercellular space to mesophyll cell cytosol, which is calculated by the C4 metabolic model (Wang *et al.*, 2014). ${Vol}_{i}$: volume of the intercellular space per leaf area (0.04 L m^-2^)

## Dynamic Leaf energy balance

For leaf energy balance, the equations used in our model were based on the method of Nikolov et al. (1995). According to this model, leaf energy balance takes account of intercepted short- and long-wave radiation, radiative energy loss from the leaf, convection, and latent heat loss in transpiration. The net photosynthesis rate *(A)*, stomata conductance and leaf temperature are inter-dependent. For example, *A* affects stomatal conductance, stomatal conductance affects leaf temperature and leaf temperature affects *A*. Instead of solving these steady-state circular connections iteratively (Nikolov et al. (1995)), differential equation describes leaf temperature (*T_leaf_*) change (Eq. 32)

$\frac{dT_{leaf}}{dt}=\frac{PAR+NIR+LR-(H+LE+E+Me)}{C_{p}\cdot m_{leaf}}$ (32)

$H=2C_{{p\_}_{air}}g_{bh}(T_{leaf}-T_{air})$ (33)

$LE=\frac{C_{lv}g_{t}}{P_{a}}{(E}_{sat}-E_{air})$ (34)

$E=2\epsilon\sigma T_{leafK}^{4}$ (35)

$M_{e}=0.506A$ (36)

Where *C_p_* is specific heat capacity of leaf, here we assumed it is the same as the specific heat capacity of water (4.184 J g^-1^ ^o^C^-1^). *m_leaf_* is the specific leaf fresh weight (g m^-2^), it was set as 198 g m^-2^ for all species based on measured value of maize leaves (197.9 ± 4.5 g m^-2^). The leaf internal air space is assumed to be saturated and the saturation vapor pressure as a function of temperature. *H* is the sensible heat, *LE* is the latent heat, *E* is the emitted long wave radiation, and *Me* is the energy of photosynthesis. The boundary layer conductance to heat is calculated as *g_bh_* = 0.924g_b_. *C_P_air_* is the specific heat capacity of air, *C_lv_* is the latent heat of vaporization of water, and g is the total conductance of the stomata and the boundary layer, $\epsilon$is the leaf emisivity of long wave radiation, $\sigma$ is the Boltzman constant.

# Model parameterization

The model took the following 11 photosynthetic parameters as input variables: maximum Rubisco activity (*V_cmax_ and f_vcmax_),* maximum PEP carboxylase activity (*V_pmax_ and f_vpmax_*), the rate constant of stomata conductance increasing and decreasing (*k_i_, k_d_*), time constant of rubisco activation (*τ_Rubisco_*), mitochondria respiration (*R_d_),* concentration of PPDK regulatory protein ([*PDRP*]), the Ball–Berry slope (*Slope_BB_*) and intercept (Intercept_BB_) (Table 4).

Then the theoretical maximal activity of PEPC (*V_max_PEPC_*) and the theoretical maximal activity of (*V_max_Rubisco_*) were calculated as:

$V_{max\_PEPC}={\frac{1}{f_{vpmax}}V}_{\mathrm{pmax}}$ (37)

$V_{max\_Rubisco}={\frac{1}{f_{vcmax}}V}_{\mathrm{cmax}}$ (38)

Based on the ratio of PEP regeneration rate and *V_cmax_* in (Von Caemmerer, 2000), the theoretical maximal activity of PPDK (*V_max_PPDK_*) and ME (*V_max_ME_*) was is assumed to be 1.33**V_cmax_.*

To estimate the enzymes activities at 25°C, temperature correction was made according to the measured temperature.

Variables and equations using input parameters are listed in the tables below (Table 4 and 5)

Table 4 The use of input variables in the dynamic C4 photosynthesis model

| Input parameter | Equation/Variable in the model | Description |
| --- | --- | --- |
| *R_d_* | *R_m_=0.5R_d_*  *R_b_=0.5R_d_* | Respiration in mesophyll cell  Respiration in bundle sheath cell |
| SlopeBB & InterceptBB | $g_{s\_steady}$ (Eq. 24) | Steady-state stomatal conductance |
| *k_i_ & k_d_* | $g_{s}$ (Eq. 23) | Transient stomatal conductance |
| [*PDRP*] | $V_{PDRP_{I}}$ (Eq. 1)  $V_{PDRP\_A}$(Eq. 2) | PPDK inactivation rate  PPDK activation rate |
| *τ_Rubisco_* | $V_{max\_Rubisco\_i}$ (Eq. 7)  *[RCA]* (Eq. 8) | Transient maximal Rubisco activity  Rubisco activase concentration |
| *V_pmax_ & f_vpmax_* | $V_{max\_PEPC}$ (Eq. 15) | Theoretical maximal PEPC activity at leaf temperature |
| *V_cmax_* | ${V_{max\_PPDK\_25}=1.33 \cdot V}_{cmax\_25}$(Eq 16) | Theoretical maximal PPDK activity at 25°C |
|  | ${V_{max\_ME\_25}=1.33 \cdot V}_{cmax\_25}$(Eq. 22) | Theoretical maximal NADP-ME activity at 25°C |
|  | ${V_{max\_MDH\_25}=1.8 \cdot V}_{cmax\_25}$(Eq. 22) | Theoretical maximal NADP-MDH activity at 25°C |
| *V_cmax_ & f_vcmax_* | $V_{m\_Rubisco}$ (Eq. 17) | Theoretical maximal Rubisco activity at leaf temperature |
|  | ${V_{max\_PE\_25}=V}_{max\_PE\_o\_meta}\cdot\frac{V_{max\_Rubisco\_25}}{V_{max\_Rubisco\_o\_meta}}$ (Eq. 22) | Except the C4 cycle enzymes and Rubisco, theoretical maximal activity of each photosynthetic enzymes in the model changes with *V_max_Rubisco_25_*.  $V_{max\_Rubisco\_o\_meta}$ is the maximum activity of Rubisco in the metabolic model (Wang et al., 2014).  $V_{max\_PE\_o\_meta}$ is the maximum activity of the photosynthetic enzyme in the metabolic model (Wang et al., 2014).  Enzyme activities are listed in Table 5 |
|  |  |  |

**Table 5** Maximum enzyme activity of C4 photosynthetic enzymes ${(V}_{max\_PE\_25})$ used in the updated dynamic model.

| **EC** | Abbreviation | Full Name | $V_{max\_PE\_o\_meta}$ (μmol m^-2^ s^-1^)  (Wang et al., 2014) | $V_{max\_PE\_25}$ (μmol m^-2^ s^-1^)  (Vmax at 25°C) |
| --- | --- | --- | --- | --- |
| **4.2.1.1** | CA | Carbonic anhydrase | 200000 | 200000 |
| **4.1.1.31** | PEPC | Phosphoenolpyruvate carboxylase | 170 | $\boldsymbol{V}_{\boldsymbol{max}\_\boldsymbol{PEPC}\_\boldsymbol{25}}$ |
| **1.1.1.82** | MDH | Malate dehydrogenase (NADP+) | 90 | $\boldsymbol{1.8\cdot V}_{\boldsymbol{cmax}\_\boldsymbol{25}}$ |
| **1.1.1.40** | ME | NADP-Malic enzyme | 90 | $\boldsymbol{1.33\cdot V}_{\boldsymbol{cmax}\_\boldsymbol{25}}$ |
| **2.7.9.1** | PPDK | Pyruvate, phosphate dikinase | 90 | $\boldsymbol{1.33\cdot V}_{\boldsymbol{cmax}\_\boldsymbol{25}}$ |
| **4.1.1.39** | Rubisco | Ribulose-bisphosphate carboxylase | 65 | $\boldsymbol{V}_{\boldsymbol{max}\_\boldsymbol{Rubisco}\_\boldsymbol{25}}$ |
| **2.7.2.3 &**  **1.2.1.13** | PGAK& GAPDH | Phosphoglycerate kinase  Glyceraldehyde-3-phosphate dehydrogenase (NADP+) | 400 | $400\cdot\frac{V_{max\_Rubisco\_25}}{65}$ |
| **4.1.2.13FBP** | Aldolase | Fructose-bisphosphate aldolase | 73.1 | $73.1\cdot\frac{V_{max\_Rubisco\_25}}{65}$ |
| **3.1.3.11** | FBPase | Fructose-bisphosphatase | 43.6 | $43.6\cdot\frac{V_{max\_Rubisco\_25}}{65}$ |
| **4.1.2.13SBP** | Aldolase | Fructose-bisphosphate aldolase | 110 | $110\cdot\frac{V_{max\_Rubisco\_25}}{65}$ |
| **3.1.3.37** | SBPase | Sedoheptulose-bisphosphatase | 29.2 | $29.2\cdot\frac{V_{max\_Rubisco\_25}}{65}$ |
| **2.2.1.1X** | Transketolase | Transketolase | 281 | $281\cdot\frac{V_{max\_Rubisco\_25}}{65}$ |
| **2.2.1.1R** | Transketolase | Transketolase | 281 | $281\cdot\frac{V_{max\_Rubisco\_25}}{65}$ |
| **2.7.1.19** | PRK | Phosphoribulokinase | 1755 | $1755\cdot\frac{V_{max\_Rubisco\_25}}{65}$ |
| **2.7.2.3M &**  **1.2.1.13M** | PGAK& GAPDH | Phosphoglycerate kinase  Glyceraldehyde-3-phosphate dehydrogenase (NADP+) | 300 | $300\cdot\frac{V_{max\_Rubisco\_25}}{65}$ |
| **4.1.2.13FBPM** | Aldolase | Fructose-bisphosphate aldolase | 8.05 | $8.05\cdot\frac{V_{max\_Rubisco\_25}}{65}$ |
| **3.1.3.11M** | FBPase | Fructose-bisphosphatase | 6.40 | $6.4\cdot\frac{V_{max\_Rubisco\_25}}{65}$ |
| **2.7.7.9** | UGPU | UTP-glucose-1-phosphate uridylyltransferase | 5.77 | $5.77\cdot\frac{V_{max\_Rubisco\_25}}{65}$ |
| **2.4.1.14** | SPS | Sucrose-phosphate synthase | 27.8 | $27.8\cdot\frac{V_{max\_Rubisco\_25}}{65}$ |
| **3.1.3.24** | SPP | Sucrose-phosphate phosphatase | 27.8 | $27.8\cdot\frac{V_{max\_Rubisco\_25}}{65}$ |
| **2.7.1.105** | PFK | 6-phosphofructo-2-kinase | 1.01 | $1.01\cdot\frac{V_{max\_Rubisco\_25}}{65}$ |
| **3.1.3.46** | F26BPP | Fructose-2,6-bisphosphate 2-phosphatase | 0.841 | $0.841\cdot\frac{V_{max\_Rubisco\_25}}{65}$ |
| **2.7.7.27** | GPA | Glucose-1-phosphate adenylyltransferase | 30 | $30\cdot\frac{V_{max\_Rubisco\_25}}{65}$ |
| **3.6.1.1** | Diphosphatase | inorganic diphosphatase | 1000 | $1000\cdot\frac{V_{max\_Rubisco\_25}}{65}$ |
| **2.4.1.21** | Starch synthase | Starch synthase | 25 | $25\cdot\frac{V_{max\_Rubisco\_25}}{65}$ |
| **4.1.1.39PR** | Rubisco | Ribulose-bisphosphate carboxylase | 65*0.11 | $Vo/Vc\_25\cdot V_{max\_Rubisco\_25}$ |
| **3.1.3.18** | PGCAP | Phosphoglycolate phosphatase | 2621 | $2621\cdot\frac{V_{max\_Rubisco\_25}}{65}$ |
| **1.1.3.15** | GO | (S)-2-hydroxy-acid oxidase &  Catalase(CAT, EC1.11.1.6) | 72.8 | $72.8\cdot\frac{V_{max\_Rubisco\_25}}{65}$ |
| **2.6.1.4** | GGAT | Glycine transaminase | 137 | $137\cdot\frac{V_{max\_Rubisco\_25}}{65}$ |
| **Gly_ser** | Gly_Ser | EC 1.4.4.2&EC2.1.2.1 | 125 | $125\cdot\frac{V_{max\_Rubisco\_25}}{65}$ |
| **2.6.1.45** | SGAT | Serine-glyoxylate transaminase | 165 | $165\cdot\frac{V_{max\_Rubisco\_25}}{65}$ |
| **1.1.1.29** | HPR | Glycerate dehydrogenase | 500 | $500\cdot\frac{V_{max\_Rubisco\_25}}{65}$ |
| **2.7.1.31** | GLYK | Glycerate kinase | 286 | $286\cdot\frac{V_{max\_Rubisco\_25}}{65}$ |
|  | **Tgca** | Glycine Transport | 300 | $300\cdot\frac{V_{max\_Rubisco\_25}}{65}$ |
|  | **Tgcea** | Glycerate Transport | 250 | $250\cdot\frac{V_{max\_Rubisco\_25}}{65}$ |
| **5.4.2.1&4.2.1.11** | M&E | Mutase and enolase | 1 | 1 or variable |
|  | JmaxM | Maximum electron transport capacity in mesophyll cell | 300 | $300\cdot\frac{V_{max\_Rubisco\_25}}{65}$ |
|  | JmaxB | Maximum electron transport capacity in bundle sheath cell | 200 | $200\cdot\frac{V_{max\_Rubisco\_25}}{65}$ |
| **3.6.3.14M** | ATPM | ATP synthase | 300 | $300\cdot\frac{V_{max\_Rubisco\_25}}{65}$ |
| **3.6.3.14B** | ATPB | ATP synthase | 300 | $300\cdot\frac{V_{max\_Rubisco\_25}}{65}$ |
| **1.18.1.2M** | NADPHM | NADP+ reductase | 200 | $200\cdot\frac{V_{max\_Rubisco\_25}}{65}$ |
| **TPTM&**  **TPTB** | TPGA  TDHAP  TGAP | PGA transport  DHAP transport  GAP transport | 750 | $750\cdot\frac{V_{max\_Rubisco\_25}}{65}$ |
| **DiT** | TOAAM | Oxaloacetate transport | 80 | $80\cdot\frac{V_{max\_Rubisco\_25}}{65}$ |
|  | TMalB/ TMalM | Malate transport | 150 | $150\cdot\frac{V_{max\_Rubisco\_25}}{65}$ |
| **PPT** | TPEPM | Phosphoenolpyruvate transport | 150 | $150\cdot\frac{V_{max\_Rubisco\_25}}{65}$ |
| **MEP** | TpyrM/ TpyrB | Pyruvate transport | 150 | $150\cdot\frac{V_{max\_Rubisco\_25}}{65}$ |

# Reference

**Ashton, A., Burnell, J. and Hatch, M.** (1984) Regulation of C4 photosynthesis: inactivation of pyruvate, Pi dikinase by ADP-dependent phosphorylation and activation by phosphorolysis. *Archives of Biochemistry and Biophysics*, **230**, 492-503.

**Ball, J., Woodrow, I. and Berry, J.** (1987) A model predicting stomatal conductance and its contribution to the control of photosynthesis under different environmental conditions. In *Progress in photosynthesis research* (Biggins, J. ed: Springer, Dordrech, pp. 221-224.

**Bernacchi, C., Pimentel, C. and Long, S.P.** (2003) In vivo temperature response functions of parameters required to model RuBP‐limited photosynthesis. *Plant, Cell & Environment*, **26**, 1419-1430.

**Boyd, R.A., Gandin, A. and Cousins, A.B.** (2015) Temperature responses of C4 photosynthesis: biochemical analysis of Rubisco, phosphoenolpyruvate carboxylase, and carbonic anhydrase in Setaria viridis. *Plant Physiology*, **169**, 1850-1861.

**Burnell, J. and Hatch, M.** (1985) Regulation of C4 photosynthesis: purification and properties of the protein catalyzing ADP-mediated inactivation and Pi-mediated activation of pyruvate, Pi dikinase. *Archives of Biochemistry and Biophysics*, **237**, 490-503.

**Chastain, C.J.** (2010) Structure, function, and post-translational regulation of C 4 pyruvate orthophosphate dikinase. In *C4 photosynthesis and related CO2 concentrating mechanisms*: Springer, pp. 301-315.

**Chastain, C.J., Baird, L.M., Walker, M.T., Bergman, C.C., Novbatova, G.T., Mamani-Quispe, C.S. and Burnell, J.N.** (2018) Maize leaf PPDK regulatory protein isoform-2 is specific to bundle sheath chloroplasts and paradoxically lacks a Pi-dependent PPDK activation activity. *Journal of experimental botany*, **69**, 1171-1181.

**Chastain, C.J., Botschner, M., Harrington, G.E., Thompson, B.J., Mills, S.E., Sarath, G. and Chollet, R.** (2000) Further analysis of maize C4 pyruvate, orthophosphate dikinase phosphorylation by its bifunctional regulatory protein using selective substitutions of the regulatory Thr-456 and catalytic His-458 residues. *Archives of Biochemistry and Biophysics*, **375**, 165-170.

**Mott, K.A. and Woodrow, I.E.** (2000) Modelling the role of Rubisco activase in limiting non‐steady‐state photosynthesis. *Journal of Experimental Botany*, **51**, 399-406.

**Roeske, C.A. and Chollet, R.** (1987) Chemical modification of the bifunctional regulatory protein of maize leaf pyruvate, orthophosphate dikinase. Evidence for two distinct active sites. *Journal of Biological Chemistry*, **262**, 12575-12582.

**Usuda, H., Ku, M.S. and Edwards, G.E.** (1984) Activation of NADP-malate dehydrogenase, pyruvate, Pi dikinase, and fructose 1, 6-bisphosphatase in relation to photosynthetic rate in maize. *Plant Physiology*, **76**, 238-243.

**Von Caemmerer, S.** (2000) *Biochemical models of leaf photosynthesis*: Csiro publishing.

**Wang, D., Portis, A.R., Moose, S.P. and Long, S.P.** (2008) Cool C4 photosynthesis: pyruvate Pi dikinase expression and activity corresponds to the exceptional cold tolerance of carbon assimilation in Miscanthus× giganteus. *Plant Physiology*, **148**, 557-567.

**Wang, Y., Long, S.P. and Zhu, X.-G.** (2014) Elements required for an efficient NADP-malic enzyme type C4 photosynthesis. *Plant physiology*, **164**, 2231-2246.

**Woodrow, I. and Mott, K.** (1989) Rate limitation of non-steady-state photosynthesis by ribulose-1, 5-bisphosphate carboxylase in spinach. *Functional Plant Biology*, **16**, 487-500.

**Woodrow, I.E. and Berry, J.** (1988) Enzymatic regulation of photosynthetic CO2, fixation in C3 plants. *Annual Review of Plant Physiology and Plant Molecular Biology*, **39**, 533-594.

**Yoshida, K. and Hisabori, T.** (2018) Determining the rate-limiting step for light-responsive redox regulation in chloroplasts. *Antioxidants*, **7**, 153.
