# Supplementary material for: Towards a dynamic photosynthesis model to guide yield improvement in C4 crops
Source: Plant J. 2021 Aug 6;107(2):343–59. doi: 10.1111/tpj.15365 (PMC9291162; doi:10.1111/tpj.15365)
Supplement: Supplementary file 3 — Appendix S2. Equations and parameters of the C4 metabolic model (Wang et al., 2014). [file TPJ-107-343-s003.docx]

**Appendix File 2 Equations and parameters of the C4 metabolic model (Wang et al., 2014)**

Contents

[1. List of Abbreviations and Their Definitions 2](#_Toc54897433)

[**1.1** **Metabolites** 2](#_Toc54897434)

[**1.2** **Enzymes and Numbering** 3](#_Toc54897435)

[**1.3** **Metabolite Transport Through Chloroplast Membrane** 5](#_Toc54897436)

[2 Equations 7](#_Toc54897437)

[**2.1** **Rate Equations** 7](#_Toc54897438)

[**2.1.1** **Light reactions** 7](#_Toc54897439)

[**2.1.2** **Metabolite transport reactions** 8](#_Toc54897451)

[**2.1.3** **CO_2_ diffusion from air space to mesophyll cell** 10](#_Toc54897472)

[**2.1.4** **C4 cycle reactions** 10](#_Toc54897474)

[**2.1.5** **Calvin Cycle reactions** 12](#_Toc54897475)

[**2.1.6** **Starch synthesis reactions** 13](#_Toc54897476)

[**2.1.7** **Sucrose synthesis reactions** 14](#_Toc54897478)

[**2.1.8** **PGA Sink reaction** 16](#_Toc54897480)

[**2.1.9** **PGA<->PEP reaction** 16](#_Toc54897482)

[**2.2.** **Differential Equations** 16](#_Toc54897486)

[**2.2.1.** **Metabolite concentration changes in mesophyll cell cytosol** 16](#_Toc54897487)

[**2.2.2.** **Metabolite concentration changes in mesophyll cell chloroplast** 17](#_Toc54897488)

[**2.2.3.** **Metabolite concentration changes in bundle sheath cell cytosol** 18](#_Toc54897489)

[**2.2.4.** **Metabolite concentration changes in bundle sheath cell chloroplast** 19](#_Toc54897490)

[**2.2.5.** **Metabolite concentration changes in bundle sheath cell peroxisome** 20](#_Toc54897491)

[**2.3** **Constants** 21](#_Toc54897492)

[**2.3.1** **Constants in mesophyll cell cytosol** 21](#_Toc54897493)

[**2.3.2** **Constants in mesophyll cell chloroplast** 21](#_Toc54897494)

[**2.3.3** **Conatants in bundle sheath cell cytosol** 22](#_Toc54897495)

[**2.3.4** **Conatants in bundle sheath cell chloroplast** 22](#_Toc54897496)

[3 Parameters 24](#_Toc54897497)

[**3.3** **Vmax of photosynthetic enzymes** 24](#_Toc54897498)

[**3.4** **Michaelis-constant and other parameters** 26](#_Toc54897499)

[5 Reference 37](#_Toc54897503)

1. **List of Abbreviations and Their Definitions**
   1. **Metabolites**

| **Abbreviation** | | **Full Name** | **Units** |
| --- | --- | --- | --- |
| ADPG | ADP-glucose | mM |  |
| Ala | Alanine | mM |  |
| Asp | Aspartate | mM |  |
| CO_2_ | Carbon dioxide | mM |  |
| CA | Total adenylate nucleotide in the chloroplast stroma including ATP and ADP | mM |  |
| CN | Total of NADP+ and NADPH in chloroplast stroma | mM |  |
| CP | The total concentration of phosphate in chloroplast stroma | mM |  |
| DHAP | Dihydroxyacetone-phosphate | mM |  |
| DPGA | 1,3-bisphosphoglycerate | mM |  |
| E4P | Erythrose 4-phosphate | mM |  |
| F26BP | Fructose 2,6–bisphosphate | mM |  |
| F6P | Fructose 6-phosphate | mM |  |
| FBP | Fructose 1,6–bisphosphate | mM |  |
| G1P | Glucose 1-phosphate | mM |  |
| G6P | Glucose 6-phosphate | mM |  |
| GAP | Glyceraldehyde 3-phosphate | mM |  |
| GCA | Glycollate | mM |  |
| GCEA | Glycerate | mM |  |
| Glu | Glutamate | mM |  |
| GLY | Glycine | mM |  |
| GOA | Glyoxylate | mM |  |
| HCO_3_ | Bicarbonate | mM |  |
| HexP | Hexose phosphate, includes F6P, G6P, and G1P | mM |  |
| HPR | Hydroxypyruvate | mM |  |
| KG | Ketoglutarate | mM |  |
| Mal | Malate | mM |  |
| O_2_ | Oxygen | mM |  |
| OAA | Oxaloacetate | mM |  |
| 2OG | 2-Oxoglutarate | mM |  |
| PEP | phosphoenolpyruvate | mM |  |
| PenP | Pentose phosphate including Ri5P, Ru5P, Xu5P | mM |  |
| PGA | 3-Phosphoglycerate | mM |  |
| PGCA | 3-Phosphoglycollate | mM |  |
| Pi | phosphate | mM |  |
| PPi | Pyrophosphate | mM |  |
| PYR | Pyruvate | mM |  |
| Ri5P | Ribose 5-phosphate | mM |  |
| Ru5P | Ribulose 5-phosphate | mM |  |
| RuBP | Ribulose 1,5-biphosphate | mM |  |
| S7P | Sedoheptulose 7-phosphate | mM |  |
| SBP | Sedoheptulose 1,7-bisphosphate | mM |  |
| SER | Serine | mM |  |
| SUC | Sucrose | mM |  |
| SUCP | Sucrose phosphate | mM |  |
| T3P | Triose phosphate including DHAP and GAP | mM |  |
| UDP | Uridine Diphosphate | mM |  |
| UDPG | Uridine Diphosphate Glucose | mM |  |
| UTP | Uridine Triphosphate | mM |  |
| Xu5P | Xylulose 5-phosphate | mM |  |

- 1. **Enzymes and Numbering**

| EC (or Model Defined ) | Abbreviation | Full Name | Numbering in the Model |
| --- | --- | --- | --- |
| 4.2.1.1 | CA | Carbonic anhydrase | 1 |
| 4.1.1.31 | PEPC | Phosphoenolpyruvate carboxylase | 2 |
| 2.7.9.1 | PPDK | Pyruvate, phosphate dikinase | 5 |
| 1.1.1.82 | MDH | Malate dehydrogenase (NADP+) | 3 |
| 1.1.1.40 | ME | NADP-Malic enzyme | 4 |
| 4.1.1.39 | Rubisco | Ribulose-bisphosphate carboxylase | 6  Pr1 |
| 2.7.2.3  2.7.2.3M | PGAK | Phosphoglycerate kinase | 7  7Mchl |
| 1.2.1.13  1.2.1.13M | GAPDH | Glyceraldehyde-3-phosphate dehydrogenase (NADP+) | 8  8Mchl |
| 5.3.1.1 | T3PI | Triose-phosphate isomerase | 9 |
| 4.1.2.13F  4.1.2.13S  4.1.2.13M | Aldolase | Fructose-bisphosphate aldolase | 10  12  Suc1 |
| 3.1.3.37 | SBPase | Sedoheptulose-bisphosphatase | 13 |
| 3.1.3.11  3.1.3.11M | FBPase | Fructose-bisphosphatase | 11  Suc2 |
| 2.2.1.1X  2.2.1.1R | Transketolase | Transketolase | 14  15 |
| 5.3.1.6 | Ri5PI | Ribose-5-phosphate isomerase | 16 |
| 5.1.3.1 | Ru5PE | Ribulose-phosphate 3-epimerase | 17 |
| 2.7.1.19 | PRK | Phosphoribulokinase | 18 |
| 5.3.1.9  5.3.1.9M | G6PI | Glucose-6-phosphate isomerase | Sta1  Suc5 |
| 5.4.2.2  5.4.2.2M | PGM | Phosphoglucomutase | Sta2  Suc6 |
| 2.7.7.27 | GPA | Glucose-1-phosphate adenylyltransferase | Sta3 |
| 3.6.1.1 | Diphosphatase | inorganic diphosphatase | Sta4 |
| 2.4.1.21 | Starch synthase | Starch synthase | Sta5 |
| 2.7.1.105M | PFK | 6-phosphofructo-2-kinase | Suc3 |
| 3.1.3.46M | F26BPP | Fructose-2,6-bisphosphate 2-phosphatase | Suc4 |
| 2.7.7.9M | UGPU | UTP-glucose-1-phosphate uridylyltransferase | Suc7 |
| 2.4.1.14M | SPS | Sucrose-phosphate synthase | Suc8 |
| 3.1.3.24M | SPP | Sucrose-phosphate phosphatase | Suc9 |
| 3.1.3.18 | PGCAP | Phosphoglycolate phosphatase | Pr2 |
| 1.1.3.15 | GO | (S)-2-hydroxy-acid oxidase &  Catalase(CAT, EC1.11.1.6) | Pr3 |
| 2.6.1.4 | GGAT | Glycine transaminase | Pr4 |
| 2.6.1.45 | SGAT | Serine-glyoxylate transaminase | Pr6 |
| 1.1.1.29 | HPR | Glycerate dehydrogenase | Pr7 |
| 2.7.1.31 | GLYK | Glycerate kinase | Pr8 |
| 3.6.1.14M  3.6.1.14B | ATPase | ATP synthase | ATPM  ATPB |
| 1.18.1.2M | FNR | Ferredoxin-NADP+ reductase | NADPHM |
| PGASink | PGASink | PGA used for amino acid synthesis or other metabolic pathway | PGASink |
| Mutase&  Enolase | Ex | 5.4.2.1&4.2.1.11 | Ex |
| Gly_ser | Gly_Ser | EC 1.4.4.2&EC2.1.2.1 | Pr5 |
| StarchDag | StarchDag | Starch degradation | StaDag |

- 1. **Metabolite Transport Through Chloroplast Membrane**

| **Model Defined**  **Numbering** | **Abbreviation** | **Full Name** |
| --- | --- | --- |
| **T_OAAB_** | T_OAAB_ (DiT2?) | Dicarboxylate transporter |
| **T_PGAM_**  **T_DHAPM_**  **T_GAPM_** | TPT M | Triose phosphate translocator in mesophyll cell, which can transport PGA DHAP and GAP. |
| **T_PGAM_**  **T_DHAPM_**  **T_GAPM_** | TPT B | Triose phosphate translocator in bundle sheath cell |
| **T_OAAM_** | DiT1 | Dicarboxylate transporter |
| **T_MALM_** |  |  |
| **T_MALB_** | DiT2 |  |
| **T_PEPM_** | PPT | PEP/phosphate translocator |
| **T_PYRM_** | MEPM | proton:pyruvate cotransporter |

1. **Equations**
   1. **Rate Equations**
      1. **Light reactions**

- - 1. **Metabolite transport reactions**

- - 1. **CO_2_ diffusion from air space to mesophyll cell**

- - 1. **C4 cycle reactions**

- - 1. **Calvin Cycle reactions**

- - 1. **Starch synthesis reactions**

- - 1. **Sucrose synthesis reactions**

- - 1. **PGA Sink reaction**

- - 1. **PGA<->PEP reaction**

2. 1. **Differential Equations**
      1. **Metabolite concentration changes in mesophyll cell cytosol**

- - 1. **Metabolite concentration changes in mesophyll cell chloroplast**

- - 1. **Metabolite concentration changes in bundle sheath cell cytosol**

- - 1. **Metabolite concentration changes in bundle sheath cell chloroplast**

- - 1. **Metabolite concentration changes in bundle sheath cell peroxisome**

- 1. **Constants**
     1. **Constants in mesophyll cell cytosol**

- - 1. **Constants in mesophyll cell chloroplast**

- - 1. **Conatants in bundle sheath cell cytosol**

- - 1. **Conatants in bundle sheath cell chloroplast**

$$[CA]_{Bchl}=[ATP]_{Bchl}+[ADP]_{Bchl}+[ADPG]_{Bchl}$$

$$[CN]_{Bchl}=[NADPH]_{Bchl}+[NADP]_{Bchl}$$

$$[GAP]_{Bchl}=\frac{K_{e\_9}\cdot[T3P]_{Bchl}}{(1+K_{e\_9})}$$

$$[DHAP]_{Bchl}=\frac{[T3P]_{Bchl}}{(1+K_{e\_9})}$$

$$[G6P]_{Bchl}=\frac{[HexP]_{Bchl}}{\frac{1}{K_{e\_Sta1}}+K_{e\_Sta2}+1}$$

$$[G1P]_{Bchl}=\frac{K_{e\_Sta2}\cdot[HexP]_{Bchl}}{\frac{1}{K_{e\_Sta1}}+K_{e\_Sta2}+1}$$

$$[F6P]_{Bchl}=\frac{\frac{[HexP]_{Bchl}}{K_{e\_Sta1}}}{\frac{1}{K_{e\_Sta1}}+K_{e\_Sta2}+1}$$

$$[Xu5P]_{Bchl}=\frac{\frac{[PenP]_{Bchl}}{K_{e\_17}}}{\frac{1}{K_{e\_16}}+\frac{1}{K_{e\_17}}+1}$$

$$[Ru5P]_{Bchl}=\frac{[PenP]_{Bchl}}{\frac{1}{K_{e\_16}}+\frac{1}{K_{e\_17}}+1}$$

$$[Ri5P]_{Bchl}=\frac{\frac{[PenP]_{Bchl}}{K_{e\_16}}}{\frac{1}{K_{e\_16}}+\frac{1}{K_{e\_17}}+1}$$

1. **Parameters**
   1. **Vmax of photosynthetic enzymes**

| **EC** | **Numbering** | **Vmax (μmol m-2 s-1)** | **Reference** |
| --- | --- | --- | --- |
| **4.2.1.1** | 1 | 200000 or variable | Hatch and Burnell (1990) with modification |
| **4.1.1.31** | 2 | 170 or variable | Kanai and Edwards (1999), Hatch (1987), von Caemmerer (2000) with modification |
| **1.1.1.82** | 3 | 90 or variable | Kanai and Edwards (1999), Hatch (1987) with modification |
| **1.1.1.40** | 4 | 90 or variable | Kanai and Edwards (1999), Hatch (1987) with modification |
| **2.7.9.1** | 5 | 90 or variable | Kanai and Edwards (1999), Hatch (1987) with modification |
| **4.1.1.39** | 6 | 65 or variable | Kanai and Edwards (1999), Hatch (1987), von Caemmerer (2000) with modification |
| **2.7.2.3 &**  **1.2.1.13** | 7 and 8 | 225 or variable | Laisk and Edwards. (2000), with modification |
| **4.1.2.13FBP** | 10 | 73.1 or variable | Zhu *et al.* (2007) with modification |
| **3.1.3.11** | 11 | 43.6 or variable | Zhu *et al.* (2007) with modification |
| **4.1.2.13SBP** | 12 | 110 or variable | Zhu *et al.* (2007) with modification |
| **3.1.3.37** | 13 | 29.2 or variable | Zhu *et al.* (2007) with modification |
| **2.2.1.1X** | 14 | 281 or variable | Zhu *et al.* (2007) with modification |
| **2.2.1.1R** | 15 | 281 or variable | Zhu *et al.* (2007) with modification |
| **2.7.1.19** | 18 | 1170 or variable | Zhu *et al.* (2007) with modification |
| **2.7.2.3M &**  **1.2.1.13M** | 7M and 8M | 300 or variable | Laisk and Edwards. (2000), with modification |
| **4.1.2.13FBPM** | Suc1 | 8.05 or variable | Zhu *et al.* (2007) with modification |
| **3.1.3.11M** | Suc2 | 6.40 or variable | Zhu *et al.* (2007) with modification |
| **2.7.7.9** | Suc7 | 5.77 or variable | Zhu *et al.* (2007) with modification |
| **2.4.1.14** | Suc8 | 27.8 or variable | Zhu *et al.* (2007) with modification |
| **3.1.3.24** | Suc9 | 27.8 or variable | Zhu *et al.* (2007) with modification |
| **2.7.1.105** | Suc3 | 1.01 or variable | Zhu *et al.* (2007) with modification |
| **3.1.3.46** | Suc4 | 0.841 or variable | Zhu *et al.* (2007) with modification |
| **2.7.7.27** | Sta3 | 30 or variable | Zhu *et al.* (2007) with modification |
| **3.6.1.1** | Sta4 | 1000 or variable | Zhu *et al.* (2007) with modification |
| **2.4.1.21** | Sta5 | 25 or variable | Zhu *et al.* (2007) with modification |
| **StarchDag** | StarchDag | 1 or 0 | Assumed |
| **PGASink** | PGASink | 2 or variable | Zhu *et al.* (2007) with modification |
| **4.1.1.39PR** | Pr1 | Vm_6*0.11 or variable | Cousins *et al*. (2010) with modification |
| **3.1.3.18** | Pr2 | 2621 or variable | Zhu *et al.* (2007) with modification |
| **1.1.3.15** | Pr3 | 72.8 or variable | Zhu *et al.* (2007) with modification |
| **2.6.1.4** | Pr4 | 137 or variable | Zhu *et al.* (2007) with modification |
| **Gly_ser** | Pr5 | 125 or variable | Zhu *et al.* (2007) with modification |
| **2.6.1.45** | Pr6 | 165 or variable | Zhu *et al.* (2007) with modification |
| **1.1.1.29** | Pr7 | 500 or variable | Zhu *et al.* (2007) with modification |
| **2.7.1.31** | Pr8 | 286 or variable | Zhu *et al.* (2007) with modification |
| **Tgca** | Pr9 | 300 or variable | Zhu *et al.* (2007) with modification |
| **Tgcea** | Pr10 | 250 or variable | Zhu *et al.* (2007) with modification |
| **5.4.2.1&4.2.1.11** | Ex | 1 or variable | Laisk and Edwards. (2000), with modification |
| **JmaxM** | JmaxM | 300 or variable | von Caemmerer (2000) with modification |
| **JmaxB** | JmaxB | JmaxM *(1-Y)/Y or variable | Assumed; Y=0.4 |
| **3.6.3.14M** | ATPM | 300 or variable | Assumed |
| **3.6.3.14B** | ATPB | 300 or variable | Assumed |
| **1.18.1.2M** | NADPHM | 200 or variable | Assumed |
| **TPTM** | TPGAM, TDHAPM, TGAPM | 750 or variable | Assumed |
| **TPTB** | TPGAM, TDHAPM, TGAPM | 750 or variable | Assumed |
| **DiT** | TOAAM | 80 or variable | Assumed |
|  | TmalB | 150 or variable | Assumed |
|  | TmalM | 150 or variable | Assumed |
| **PPT** | TPEPM | 150 or variable | Assumed |
| **MEPM** | TpyrM | 150 or variable | Assumed |
| **MEPB** | TpyrB | 150 or variable | Assumed |

- 1. **Michaelis-constant and other parameters**

| **EC** | | | **Numbering** | | | **parameters** | **Reference** |  |  |
| --- | --- | --- | --- | --- | --- | --- | --- | --- | --- |
| **Inf** | | | Inf | | | g_m_=0.7 mol m^-2^ bar^-1^ | Assumed |  |  |
|  | | |  | | | Sc= 0.33 × 10^-4^  mmol L^-1^μbar^-1^ | Uchida *et al.* (1983), Hoofd *et al.* (1986) |  |  |
| **4.2.1.1** | | | 1 | | | K_mCO2_1_=2.8 mM | Hatch and Burnell (1990) |  |  |
|  | | |  | | | K_mHCO3_1_=34 mM | Pocker and Miksch (1978) |  |  |
|  | | |  | | | [H+]=10e-7.3mM | Felle and Bertl (1986) |  |  |
|  | | |  | | | K_e_1_ = 5.6e-7mM | Pocker and Miksch (1978) |  |  |
|  | | |  | | |  |  |  |  |
| **4.1.1.31** | | | 2 | | | K_mHCO3_2_ =0.02 mM | Uedan and Sugiyama (1976) |  |  |
|  | | |  | | | K_mPEP_2_ = 0.1 mM | Mukerji (1977) |  |  |
|  | | |  | | | K_imal_2_ =0. 5 mM | Gao and Woo (1996) |  |  |
|  | | |  | | |  |  |  |  |
| **1.1.1.82** | | | 3 | | | K_mNADPH_3_ =0.024 mM | Kagawa and Bruno (1988) |  |  |
|  | | |  | | | K_mOAA_3_ =0.056 mM | Kagawa and Bruno (1988) |  |  |
|  | | |  | | | K_mNADP_3_ =0.073 mM | Kagawa and Bruno (1988) |  |  |
|  | | |  | | | K_mmal_3_ =32.0 mM | Kagawa and Bruno (1988) |  |  |
|  | | |  | | | K_e_3_ =4450.0 | Laisk and Edwards (2000) |  |  |
|  | | |  | | |  |  |  |  |
| **1.1.1.40** | | | 4 | | | K_mCO2_4_ =1.1 mM | Jenkins *et al.* (1987) |  |  |
|  | | |  | | | K_mNADP_4_ =0.0080 mM | Detarsio *et al.* (2003) |  |  |
|  | | |  | | | K_mNADPH_4_ =0.045 mM | Ziegler (1974) |  |  |
|  | | |  | | | K_mPyr_4_ =3.0 mM | Ziegler (1974) |  |  |
|  | | |  | | | K_mmal_4_ =0.23 mM | Detarsio *et al.* (2003) |  |  |
|  | | |  | | | K_e_4_ =0.051 mM | Harary *et al.* (1953) |  |  |
|  | | |  | | |  |  |  |  |
| **2.7.9.1** | | | 5 | | | K_iPEP_5_ =0.15 mM | Jenkins and Hatch (1985) |  |  |
|  | | |  | | | K_mATP_5_ =0.082 mM | Jenkins and Hatch (1985) |  |  |
|  | | |  | | | K_mPyr_5_ =0.082 mM | Jenkins and Hatch (1985) |  |  |
|  | | |  | | |  |  |  |  |
| **4.1.1.39** | | | 6 | | | K_mCO2_6_ =0.0162 mM | Cousins *et al.* (2010) |  |  |
|  | | |  | | | K_mO2_6_ =0.222 mM | Cousins *et al.* (2010) |  |  |
|  | | |  | | | K_mRuBP_6_ =0.02 mM | Farquhar (1979) |  |  |
|  | | |  | | | K_iPGA_6_ =2.52 mM | Assumed, Badger and Lorimer (1981) |  |  |
|  | | |  | | | K_iFBP_6_ =0.04 mM | Badger and Lorimer (1981) |  |  |
|  | | |  | | | K_iSBP_6_ =0.75 mM | Badger and Lorimer (1981) |  |  |
|  | | |  | | | K_iPi_6_ =3.6 mM | Assumed, Badger and Lorimer (1981) |  |  |
|  | | |  | | | K_iNADPH_6_ =0.21 mM | Assumed, Badger and Lorimer (1981) |  |  |
|  | | |  | | |  |  |  |  |
| **2.7.2.3 &**  **1.2.1.13** | | | 7 and 8 | | | K_mPGA_78_=1 mM | Laisk and Edwards (2000) |  |  |
|  |  |  |  | | | K_mATP_78_=0.3 mM | Laisk and Edwards (2000) |  |  |
|  | | |  | | | K_mNADPH_78_=0.05 mM | Ferri *et al.*(1978), Trost (1993), Macioszek and Anderson (1987), Baalmann *et al.*, 1995, Sparla *et al.*, (2004), Sparla *et al.*, (2005), |  |  |
|  | | |  | | |  |  |  |  |
| **5.3.1.1** | | | 9 | | | K_e_9_=0.05 | Bassham and Krause (1969) |  |  |
|  | | |  | | |  |  |  |  |
| **4.1.2.13FBP** | | | 10 | | | K_mDHAP_10_ =0.4 mM | Iwaki *et al.* (1991) |  |  |
|  | | |  | | | K_mGAP_10_ =0.3 mM | Iwaki *et al.* (1991), Zhu *et al.* (2007) |  |  |
|  | | |  | | | K_mFBP_10_ =0.02 mM | Brooks and Criddle (1966), Schnarrenberger and Kruger (1986) |  |  |
|  | | |  | | | K_e_10_ =7.1 mM^-1^ | Bassham and Krause (1969),  Iwaki *et al.* (1991) |  |  |
|  | | |  | | |  |  |  |  |
| **3.1.3.11** | | | 11 | | | K_iF6P_11_ =0.7 mM | Heldt (1983) |  |  |
|  | | |  | | | K_iPi_11_ =12.0 mM | Charles and Halliwell (1981) |  |  |
|  | | |  | | | K_mFBP_11_ =0.033 mM | Charles and Halliwell (1981) |  |  |
|  | | |  | | | K_e_11_ =666000.0 mM | Bassham and Krause (1969) , Laisk *et al.*(1989) |  |  |
|  | | |  | | |  |  |  |  |
| **4.1.2.13SBP** | | | 12 | | | K_mDHAP_12_ =0.4 mM | Iwaki *et al.* (1991) |  |  |
|  | | |  | | | K_mE4P_12_ =0.2 mM | Zhu *et al.* (2007) |  |  |
|  | | |  | | | K_mSBP_12_=0.02 mM | Brooks and Criddle (1966) |  |  |
|  | | |  | | | K_e_12_ =1.017 mM^-1^ | Bassham and Krause (1969) , Laisk *et al.*(1989) |  |  |
|  | | |  | | |  |  |  |  |
| **3.1.3.37** | | | 13 | | | K_iPi_13_ =12.0 mM | Woodrow *et al.* (1983) |  |  |
|  | | |  | | | K_mSBP_13_ =0.05 mM | Woodrow *et al.* (1983), Cadet and Meunier (1988) |  |  |
|  | | |  | | | K_e_13_ =666000.0 mM | Bassham and Krause (1969), Laisk *et al.*(1989) |  |  |
|  | | |  | | |  |  |  |  |
| **2.2.1.1X** | | | 14 | | | K_mE4P_14_ =0.1 mM | Zhu *et al.* (2007) |  |  |
|  | | |  | | | K_mF6P_14_ =0.1 mM | Zhu *et al*. (2007) |  |  |
|  | | |  | | | K_mGAP_14_ =0.1 mM | Sprenger *et al.* (1995), Schenk *et al.* (1998), Zhu *et al*. (2007) |  |  |
|  | | |  | | | K_mXu5P_ =0.1 mM | Schenk *et al.* (1998), Laisk *et al.* (1989), Zhu *et al*. (2007) |  |  |
|  | | |  | | | K_e_14_ =0.084 | Datta *et al*. (1961). |  |  |
| **2.2.1.1R** | | | 15 | | | K_mGAP_15_ =0.072 mM | Albe (1991); Laisk *et al.* (1989) |  |  |
|  | | |  | | | K_mRi5P_15_ =1.5 mM | Albe (1991); Laisk *et al.* (1989) |  |  |
|  | | |  | | | K_mS7P_15_ =0.46 mM | Albe (1991); Laisk *et al.* (1989) |  |  |
|  | | |  | | | K_mXu5P_15_ =0.1 mM | Albe (1991); Laisk *et al.* (1989) |  |  |
|  | | |  | | | K_e_15_ =1.176 | Bassham and Krause (1969), Laisk *et al.* (1989) |  |  |
| **5.3.1.6** | | | 16 | | | K_e_16_=0.4 | Bassham and Krause (1969) |  |  |
| **5.1.3.1** | | | 17 | | | K_e_17_=0.67 | Bassham and Krause, (1969) |  |  |
| **2.7.1.19** | | | 18 | | | K_iADP_18_ =2.5 mM | Gardemann *et al.* (1983) |  |  |
|  | | |  | | | K_i_ADP_18_ =0.4 mM | Gardemann *et al.* (1983) |  |  |
|  | | |  | | | K_iPGA_18_ =2.0 mM | Gardemann *et al.* (1983) |  |  |
|  | | |  | | | K_iPi_18_ =4.0 mM | Gardemann *et al.* (1983) |  |  |
|  | | |  | | | K_iRuBP_18_ =0.7 mM | Gardemann *et al.* (1983) |  |  |
|  | | |  | | | K_mATP_18_ =0.625 mM | Slabas *et al.* (1976) |  |  |
|  | | |  | | | K_mRu5P_18_ =0.05 mM | Gardemann *et al.* (1983), Omnaas *et al.*(1985) |  |  |
|  | | |  | | | K_e_18_ =6846.0 | Bassham and Krause (1969), Laisk *et al.* (1989) |  |  |
|  | | |  | | |  |  |  |  |
| **4.1.2.13FBPM** | | | Suc1 | | | K_mDHAP_Suc1_=0.45 mM | Iwaki *et al.* (1991) |  |  |
|  | | |  | | | K_mGAP_Suc1_ =0.04 mM | Iwaki *et al.* (1991) |  |  |
|  | | |  | | | K_mFBP_Suc1_=0.023 mM | Schnarrenberger (1986) |  |  |
|  | | |  | | | K_e_Suc1_ =12.0 mM^-1^ | Thomas *et al.* (1997), Zhu *et al*. (2007) |  |  |
|  | | |  | | |  |  |  |  |
| **3.1.3.11M** | | | Suc2 | | | K_iF26BP_Suc2_ =0.007mM | Jang *et al.* (2003) |  |  |
|  | | |  | | | K_iF6P_Suc2_ =0.7 mM | Heldt *et al.* (1983) |  |  |
|  | | |  | | | K_iPi_Suc2_ =12.0 mM | Charles & Halliwell (1981) |  |  |
|  | | |  | | | K_mFBP_Suc2_ =0.0025mM | Jang *et al.* (2003) |  |  |
|  | | |  | | | K_e_Suc2_ =174.0 mM | Lawson *et al.* (1976) |  |  |
|  | | |  | | |  |  |  |  |
| **5.3.1.9M** | | | Suc5 | | | K_e_Suc5_=2.3 | Bassham and Krause (1969), |  |  |
|  | | |  | | |  |  |  |  |
| **5.4.2.2M** | | | Suc6 | | | K_e_Suc6_=0.0584 | Bassham and Krause (1969), |  |  |
|  | | |  | | |  |  |  |  |
| **2.7.7.9** | | | Suc7 | | | K_mG1P_Suc7_ =0.14 mM | Nakano *et al.* (1989) |  |  |
|  | | |  | | | K_mPPi_Suc7_ =0.11 mM | Nakano *et al.* (1989) |  |  |
|  | | |  | | | K_mUDPG_Suc7_ =0.12mM | Nakano *et al.* (1989) |  |  |
|  | | |  | | | K_mUTP_Suc7_ =0.1 mM | Nakano *et al.* (1989) |  |  |
|  | | |  | | | K_e_Suc7_ =0.31 mM | Hansen *et al.* (1966). |  |  |
|  | | |  | | |  |  |  |  |
| **2.4.1.14** | | | Suc8 | | | K_iFBP_Suc8_ =0.8 mM | Harbron *et al.* (1981) |  |  |
|  | | |  | | | K_iPi_Suc8_ =11.0 mM | Harbron *et al.* (1981) |  |  |
|  | | |  | | | K_iSuc_Suc8_ =50.0 mM | Salermo and Pontis (1978) |  |  |
|  | | |  | | | K_iSucP_Suc8_ =0.4 mM | Harbron *et al.* (1981) |  |  |
|  | | |  | | | K_iUDP_Suc8_ =0.7 mM | Harbron *et al.* (1981) |  |  |
|  | | |  | | | K_mF6P_Suc8_ =0.8 mM | Lunn and Rees (1990) |  |  |
|  | | |  | | | K_mUDPG_Suc8_ =2.4 mM | Lunn and Rees (1990) |  |  |
|  | | |  | | | K_e_Suc8_ =10.0 | Lunn and Rees (1990) |  |  |
|  | | |  | | |  |  |  |  |
| **3.1.3.24** | | | Suc9 | | | K_mSuc_Suc9_ =80.0 mM | Cumino (2001) |  |  |
|  | | |  | | | K_mSucP_Suc9_ =0.35 mM | Whitaker (1984) |  |  |
|  | | |  | | | K_e_Suc9_ =780.0 | Zhu *et al*. (2007) |  |  |
|  | | |  | | |  |  |  |  |
| **2.7.1.105** | | | Suc3 | | | K_iADP_Suc3_ =0.16 mM | Kretschmer and Hofmann (1984) |  |  |
|  | | |  | | | K_iDHAP_Suc3_ =0.7 mM | Markham and Kruger (2002) |  |  |
|  | | |  | | | K_mATP_Suc3_ =0.5 mM | Walker and Huber (1987), Markham and Kruger (2002) |  |  |
|  | | |  | | | K_mF26BP_Suc3_ =0.021 mM | Garcia de Frutos and Baanante (1995) |  |  |
|  | | |  | | | K_mF6P_Suc3_ =0.5 mM | Walker and Huber (1987), Markham and Kruger (2002) |  |  |
|  | | |  | | | K_e_Suc3_ =590.0 | Cornish-Bowden (1997) |  |  |
|  | | |  | | |  |  |  |  |
| **3.1.3.46** | | | Suc4 | | | K_iF6P_Suc4_ =0.1 mM | Villadsen and Nielsen (2001) |  |  |
|  | | |  | | | K_iPi_Suc_4 =0.5 mM | Villadsen and Nielsen (2001) |  |  |
|  | | |  | | | K_mF26BP_Suc4_= 0.032 mM | Macdonald *et al*. (1989) |  |  |
| **5.3.1.9** | | | Sta1 | | | K_e_Sta1_=2.3 | Bassham and Krause (1969) |  |  |
|  | | |  | | |  |  |  |  |
| **5.4.2.2** | | | Sta2 | | | K_e_Sta2_=0.058 | Colowick and Sutherland (1942) |  |  |
|  | | |  | | |  |  |  |  |
| **2.7.7.27** | | | Sta3 | | | K_aPGA_Sta1_=0.2252 mM | Assumed |  |  |
|  | | |  | | | K_mG1P_Sta3_=0.038 mM | Fuchs *et al*. (1979) |  |  |
|  | | |  | | | K_mATP_Sta3_=0.12 mM | Boehlein *et al*. (2005) |  |  |
|  | | |  | | | K_iPi_ATP_Sta3_=2.96 mM | Boehlein *et al*. (2005) |  |  |
|  | | |  | | | K_mPPi_Sta3_=0.033 mM | Amir *et al*. (1972) |  |  |
|  | | |  | | | K_iCPP1_ATP_Sta3_=13.8E-4 mM | Amir *et al*. (1972) |  |  |
|  | | |  | | | K_mADPG_Sta3_=0.24 mM | Sowokinos (1981) |  |  |
|  | | |  | | | K_iADP_ATP_Sta3_=2.0 mM | Ghosh *et al*. (1966) |  |  |
|  | | |  | | | K_e_Sta3_=1.1 | Espada (1962) |  |  |
|  | | |  | | |  |  |  |  |
| **3.6.1.1** | | | Sta4 | | | K_mPPi_Sta2_=0.154 mM | Van *et al*. (2005) |  |  |
|  | | |  | | | K_e_Sta2_=1.57 E-4 mM | Flodgaard *et al*. (1974) |  |  |
| **2.4.1.21** | | | Sta5 | | | K_mADPG_Sta3_=0.077 mM | Hawker *et al*. (1974) |  |  |
|  | | |  | | |  |  |  |  |
| **PGASink** | | | PGAsink | | | K_mPGA_PGASink_=2.4 mM | assumed |  |  |
|  | | |  | | |  |  |  |  |
| **4.1.1.39PR** | | | PR1 | | | K_mCO2_PR1_=0.0162 mM | Cousins *et al.* (2010) |  |  |
|  | | |  | | | K_mO2_PR1_=0.222 mM | Cousins *et al.*(2010) |  |  |
|  | | |  | | | K_mRuBP_PR1_=0.02 mM | Farquhar (1979) |  |  |
|  | | |  | | | K_iPGA_PR1_=2.52 mM | Assumed Badger and Lorimer (1981) |  |  |
|  | | |  | | | K_iFBP_PR1_=0.04 mM | Badger and Lorimer (1981) |  |  |
|  | | |  | | | K_iSBP_PR1_=0.75 mM | Badger and Lorimer (1981) |  |  |
|  | | |  | | | K_iPi_PR1_=3.6 mM | Assumed Badger and Lorimer (1981) |  |  |
|  | | |  | | | K_iNADPH_PR1_=0.21 mM | Assumed Badger and Lorimer (1981) |  |  |
|  | | |  | | |  |  |  |  |
| **3.1.3.18** | | | PR2 | | | K_mPGCA_PR2_=0.026 mM | Christeller and Tolbert (1978) |  |  |
|  | | |  | | | K_iPI_PR2_=2.55 mM | Christeller and Tolbert (1978) |  |  |
|  | | |  | | | K_iGCA_PR2_=94.0 mM | Christeller and Tolbert (1978) |  |  |
|  | | |  | | |  |  |  |  |
| **1.1.3.15** | | | PR3 | | | K_mGCA_PR3_= 0.1 mM | Tolbert (1981) |  |  |
|  | | |  | | |  |  |  |  |
| **2.6.1.4** | | | PR4 | | | K_e_PS4_= 607.0 | Cooper and Meister (1972) |  |  |
|  | | |  | | | K_mGOA_PS4_=0.15 mM | Nakamura and Tolbert (1983) |  |  |
|  | | |  | | | K_mGLU_PS4_= 1.7 mM | Nakamura and Tolbert (1983) |  |  |
|  | | |  | | | K_iGLY_PS4_=2.0 mM | Zhu et al (2007) |  |  |
|  | | |  | | |  |  |  |  |
| **2.6.1.45** | | | PR6 | | | K_e_PR6_= 0.24 | Guynn (1982) |  |  |
|  | | |  | | | K_mGOA_PR6_=0.15 mM | Nakamura and Tolbert (1983) |  |  |
|  | | |  | | | K_mSER_PR6_=2.7 mM | Nakamura and Tolbert (1983) |  |  |
|  | | |  | | | K_iGLY_PR6_=33.0 mM | Nakamura and Tolbert (1983) |  |  |
|  | | |  | | |  |  |  |  |
| **1.1.1.29** | | | PR7 | | | K_e_PR7_= 2.5 E-5 | Guynn (1982), Zhu et al (2007) |  |  |
|  | | |  | | | K_iHPR_PR7_= 12.0 mM | Kleczkowski and Edwards (1989) |  |  |
|  | | |  | | | K_mHPR_PR7_=0.09 mM | Kleczkowski and Edwards (1989) |  |  |
|  | | |  | | |  |  |  |  |
| **2.7.1.31** | | | PR8 | | | K_e_PR8_= 300.0 | Kleczkowski *et al.* (1985) |  |  |
|  | | |  | | | K_mATP_PR8_= 0.21 mM | Kleczkowski *et al.* (1985) |  |  |
|  | | |  | | | K_mGCEA_PR8_=0.25 mM | Kleczkowski *et al.* (1985) |  |  |
|  | | |  | | | K_iPGA_PR8_=0.72 mM | Assumed |  |  |
|  | | |  | | |  |  |  |  |
| **Gly_ser** | | | PR5 | | | K_mGLY_PS5_= 6.0 mM | Douce *et al.* (2001) |  |  |
|  | | |  | | | K_iSER_PS5_=4.0 mM | Douce *et al.* (2001) |  |  |
| **Tgca** | | | PR9 | | | K_mGCA_PR9_= 0.2 mM | Howitz and McCarty (1985) |  |  |
|  | | |  | | | K_iGCEA_PR9_= 0.22 mM | Howitz and McCarty (1985) |  |  |
|  | | |  | | |  |  |  |  |
| **Tgcea** | | | PR10 | | | K_mGCEA_PR10_= 0.39mM | Howitz and McCarty (1986) |  |  |
|  | | |  | | | K_iGCA_PR10_= 0.28 mM | Howitz and McCarty (1986) |  |  |
|  | | |  | | |  |  |  |  |
| **5.4.2.1&4.2.1.11** | | | Ex | | | K_mPGA_62_ = 0.1 mM | Laisk and Edwards (2000) |  |  |
|  | | |  | | | K_mPEP_62_ = 0.5 mM | Laisk and Edwards (2000) |  |  |
|  | | |  | | | Ke_62=0.4302 | Laisk and Edwards (2000) |  |  |
|  | | |  | | |  |  |  |  |
| **3.6.3.14M** | | | ATPM | | | K_mADP_ATPM_ =0.014 mM | Davenport and Mccarty (1986) |  |  |
|  | | |  | | | K_mATP_ATPM_ =0.11 mM | Penefsky (1974) |  |  |
|  | | |  | | | K_mPi_ATPM_ =0.3 mM | Aflalo and Shavit (1983) |  |  |
|  | | |  | | | K_e_ATPM_ =5.734 mM-1 | Bassham and Krause (1969), Laisk *et al.*(1989) |  |  |
|  | | |  | | | X =0.667 | Assumed light partition coefficient |  |  |
|  | | |  | | | Y =0.6 | Assumed Jmax partition coefficient |  |  |
|  | | |  | | | F =0.7225 | von Caemmerer (2000) F=abs(1-f) |  |  |
|  | | |  | | | θ=0.7 | von Caemmerer (2000) |  |  |
|  | | |  | | | D =1 | von Caemmerer (2000) ATP/e-  whole chain +Qcycle |  |  |
|  | | |  | | |  |  |  |  |
| **3.6.3.14B** | | | ATPB | | | K_mADP_ATPB_ =0.014 mM | Davenport and Mccarty (1986) |  |  |
|  | | |  | | | KmPi_ATPB =0.11 mM | Penefsky (1974) |  |  |
|  | | |  | | | KmATP_ATPB =0.3 mM | Aflalo and Shavit (1983) |  |  |
|  | | |  | | | K_e_ATPB_ =5.734 mM^-1^ | Bassham and Krause (1969), Laisk et al.(1989) |  |  |
|  | | |  | | | G =0.667 | von Caemmerer (2000) ATP/e- |  |  |
|  | | |  | | |  |  |  |  |
| **1.18.1.2M** | | | NADPHM | | | K_mNADP_NADPHM_=0.0072 mM | Shin (1972) |  |  |
|  | | |  | | | K_mNADPH_NADPHM_=0.036 mM | Gozzer *et al*. (1977) |  |  |
|  | | |  | | | K_e_NADPHM_ =502 | Knaff (1996), Keirns (1972), Laisk and Edwards (2000) |  |  |
|  | | |  | | | E =0.5 | von Caemmerer (2000) |  |  |
| **1.18.1.2M** | | | NADPHB | | | K_mNADP_NADPHM_=0.0072 mM | | Shin (1972) |  |
|  | | |  | | | K_mNADPH_NADPHM_=0.036 mM | | Gozzer *et al*. (1977) |  |
|  | | |  | | | K_e_NADPHM_ =502 | | Knaff (1996), Keirns (1972), Laisk and Edwards (2000) |  |
|  | | |  | | | E =0.5 | | von Caemmerer (2000) |  |
|  | | |  | | | u | | Assumed light partition coefficient of linear electron transport in BSC |  |
|  | | |  | | | v | | Assumed Jmax partition coefficient of linear electron transport in BSC |  |
| **Metabolite transport through Plasmodesmata** | | | Leak | | | D_CO2_PD_=1.7×10-9 | Evans *et al.* (2009) |  |  |
|  |  |  |  | | | l_PD_=0.4μm | von Caemmerer and Furbank (2003) |  |  |
|  |  |  |  | | | S_W_/S_l_=0.83 | Sowinski *et al.* (2008) |  |  |
|  |  |  |  | | | φ=0.03 | Assumed |  |  |
|  |  |  | TMAL | | | D_MAL_PD_=6.67×10^-10^ | Sowinski et al (2008) |  |  |
|  |  |  | TPYR | | | D_PYR_PD_=7.00×10^-10^ | Sowinski et al (2008) |  |  |
|  |  |  | TPGA | | | D_PGA_PD_=5.25×10^-10^ | Sowinski et al (2008) |  |  |
|  |  |  | TGAP | | | D_GAP_PD_=5.25×10^-10^ | Sowinski et al (2008) |  |  |
|  |  |  | TDHAP | | | D_DHAP_PD_=5.25×10^-10^ | Sowinski et al (2008) |  |  |
|  | | | TAsp | | | D_Asp_PD_=6.40×10^-10^ | | | Sowinski et al (2008) |
|  |  |  | TAla | | | D_Ala_PD_=8.40×10^-10^ | | | Sowinski et al (2008) |
|  |  |  | TPEP | | | D_PEP_PD_=6.30×10^-10^ | | | Sowinski et al (2008) |
|  | | | TO_2_ | | | D_O2_PD_=1.8×10-9 | | | Assumed, according to D_CO2_PD_ |
| **Leak_Bchl** | | | Leak_Bchl | | | P_CO2_B_=0.002 cm s^-1^ | Evans et al (2009)  Uehlein et al. (2008) |  |  |
|  | | | TO2_Bchl | | | P_O2_B_=0.002 cm s^-1^ | Assumed, according to P_CO2_B_ |  |  |
|  | | |  | | | S_Chl_/S_l_ =10 | Assumed |  |  |
| **DiT1** | | | TOAAM | | | K_m_OAA_M_=0.053 | Hatch *et al.* (1984) |  |  |
|  | | |  | | | K_imal_OAA_M_=7.5 | Hatch *et al.* (1984) |  |  |
|  | | | TmalM | | | K_m_MAL_M_=0.5 | Day and Hatch (1981) |  |  |
|  | | |  | | | K_iOAA_MAL_M_=0.3 | Day and Hatch (1981) |  |  |
| **DiT2** | | | TmalB | | | K_m_MAL_B_=1 | Assumed |  |  |
| **PPT** | | | TPEPM | | | K_m_PEP_M_=0.3 | Assumed |  |  |
| **MEPM** | | | TpyrM | | | K_m_PYR_M_=0.05 | Assumed |  |  |
| **MEPB** | | | TpyrB | | | K_m_PYR_B_=0.05 | Assumed |  |  |
| **TPTM** | | | TPGAM | | | K_mPGA_ = 2 | Assumed |  |  |
|  |  |  | TGAPM | | | K_mGAP_ = 2 | Assumed |  |  |
|  |  |  | TDHAPM | | | K_mDHAP_ = 2 | Assumed |  |  |
| **TPTB** | | | TPGAB | | | K_mPGA_ = 2 | Assumed |  |  |
|  | | | TGAPB | | | K_mGAP_ = 2 | Assumed |  |  |
|  | | | TDHAPB | | | K_mDHAP_ = 2 | Assumed |  |  |

4. **Reference**

**Aflalo C, Shavit N** (1983) Steady­state kinetics of photophosphorylation: limited access of nucleotides to the active site on the ATP synthetase. FEBS Lett. 154: 1 75-79

**Agostino A, Jeffrey P, Hatch MD** (1992) Amino Acid Sequence and Molecular Weight of Native NADP Malate Dehydrogenase from the C(4) Plant Zea mays. Plant Physiol 98: 1506-1510

**Albe KR** (1991) Partial puriﬁcation and kinetic characterization of transaldolase from Dictyostelium discoideum. Exp Mycol 15:255–62

**Amir J, Cherry JH** (1972) Purification and properties of adenosine diphosphoglucose pyrophosphorylase from sweet corn. Plant Physiol 49: 893-897

**Baalmann E, Backhausen JE, Rak C, Vetter S, Scheibe R** (1995) Reductive modification and nonreductive activation of purified spinach chloroplast NADP-dependent glyceraldehyde-3-phosphate dehydrogenase. Arch Biochem Biophys 324: 201-208

**Bassham JA, Krause GH** (1969). Free energy changes and metabolic regulation in steady-state photosynthetic carbon reduction. Biochim Biophys Acta 189: 207-221

**Badger MR, Lorimer GH** (1981) Interaction of sugar phosphates with the catalytic site of ribulose-1,5-bisphosphate carboxylase. Biochemistry-Us 20: 2219-2225

**Baez M, Rodriguez PH, Babul J, Guixe V** (2003) Structural and functional roles of Cys-238 and Cys-295 in Escherichia coli phosphofructokinase-2. Biochem J 376: 277-283

**Bassham JA, Krause GH** (1969) Free energy changes and metabolic regulation in steady-state photosynthetic carbon reduction. Biochim Biophys Acta 189: 207-221

**Bentahir M, Feller G, Aittaleb M, Lamotte-Brasseur J, Himri T, Chessa JP, Gerday C** (2000) Structural, kinetic, and calorimetric characterization of the cold-active phosphoglycerate kinase from the antarctic Pseudomonas sp. TACII18. J Biol Chem 275: 11147-11153

**Brooks K, Criddle RS** (1966) Enzymes of the carbon cycle of photosynthesis. I. Isolation and properties of spinach chloroplast aldolase. Arch Biochem Biophys 117:650–659

**Boehlein SK, Sewell AK, Cross J, Stewart JD, Hannah LC** (2005) Purification and characterization of adenosine diphosphate glucose pyrophosphorylase from maize/potato mosaics. Plant Physiol 138: 1552-1562

**Cadet F, Meunier JC** (1988) pH and kinetic studies of chloroplast sedoheptulose-1,7 -bisphosphatase from spinach (Spinacia oleracea). Biochem J 253: 249-254

**Cadet F, Meunier JC, Ferte N** (1987) Isolation and purification of chloroplastic spinach (Spinacia oleracea) sedoheptulose-1,7-bisphosphatase. Biochem J 241: 71-74

**Charles SA, Halliwell B** (1981) Light activation of fructose bisphosphatase in photosynthetically competent pea chloroplasts. Biochem J 200: 357-363

**Chen ZH, Walker RP, Acheson RM, Leegood RC** (2002) Phosphoenolpyruvate carboxykinase assayed at physiological concentrations of metal ions has a high affinity for CO_2_

 Plant Physiol. 128, 160-164

**Christeller JT, Tolbert NE** (1978) Phosphoglycolate phosphatase. Purification and properties. J Biol Chem 253: 1780-1785

**Colowick SP, Sutherland EW** (1942) Polysaccharide synthesis from glucose by means of purified enzymes. J Biol Chem 144: 423-437

**Cooper JL, Meister A** (1972) Isolation and properties of highly purified glutamine transaminase. Biochemistry-Us 11: 661-671

**Cousins AB, Ghannoum O, Von Caemmerer S, Badger MR** (2010) Simultaneous determination of Rubisco carboxylase and oxygenase kinetic parameters in Triticum aestivum and Zea mays using membrane inlet mass spectrometry. Plant Cell Environ 33: 444-452

**Cumino A, Ekeroth C, Salerno GL** (2001) Sucrose-phosphate phosphatase from Anabaena sp. strain PCC 7120: isolation of the protein and gene revealed significant structural differences from the higher-plant enzyme. Planta 214: 250-256

**Datta AG, Racker E** (1961) Mechanism of action of transketolase. I. Properties of the crystalline yeast enzyme. J Biol Chem 236: 617-623

**Davenport JW, McCarty RE** (1986) Relationships between rates of steady-state ATP synthesis and the magnitude of the protonactivity gradient across thylakoid membranes. Biochim Biophys Acta 851: 136–145

**Day DA, Hatch MD** (1981) Dicarboxylate Transport in Maize Mesophyll Chloroplasts. Arch Biochem Biophys 211: 738-742

**Detarsio E, Wheeler MC, Campos Bermudez VA, Andreo CS, Drincovich MF** (2003) Maize C4 NADP-malic enzyme. Expression in Escherichia coli and characterization of site-directed mutants at the putative nucleoside-binding sites. J Biol Chem 278: 13757-13764

**Dietz KJ, Heber U** (1984) Rate-limiting factors in leaf photosynthesis. 1. Carbon fluxes in the Calvin cycle. Biochim Biophys Acta 767: 432–443

**Douce R, Bourguignon J, Neuburger M, Rebeille F** (2001) The glycine decarboxylase system: a fascinating complex. Trends Plant Sci 6: 167-176

**Echeverria E, Salerno G** (1994) Properties of sucrose-phosphate phosphatase from rice (Oryza sativa) leaves. Plant Sci. 96, 15-19

**Espada J** (1962) Enzymic synthesis of adenosine diphosphate glucose from glucose-l-phosphate and adenosine triphosphate. J Biol Chem 237:3577-3581

**Evans JR, Kaldenhoff R, Genty B, Terashima I** (2009) Resistances along the CO2 diffusion pathway inside leaves. J Exp Bot 60: 2235-2248

**Farquhar GD** (1979) Models describing the kinetics of ribulose biphosphate carboxylase-oxygenase. Arch Biochem Biophys 193: 456-468

**Felle H, Bertl A** (1986) The Fabrication Of H+-Selective Liquid-Membrane Microelectrodes for Use In Plant-Cells. J Exp Bot 37: 1416-1428

**Ferri G, Comerio G, Iadarola P, Zapponi MC, Speranza ML** (1978) Subunit structure and activity of glyceraldehyde-3-phosphate dehydrogenase from spinach chloroplasts. Biochim Biophys Acta 522: 19-31

**Fifis T, Scopes RK** (1978) Purification of 3-phosphoglycerate kinase from diverse sources by affinity elution chromatography. Biochem J 175: 311-319

**Flodgaard H, Fleron P** (1974) Thermodynamic parameters for the hydrolysis of inorganic pyrophosphate at pH 7.4 as a function of (Mg2+), (K+), and ionic strength determined from equilibrium studies of the reaction. J Biol Chem 249: 3465-3474

**Fuchs RL, Smith JD** (1979) The purification and characterization of ADP-glucose pyrophosphorylase A from developing maize seeds. Biochim Biophys Acta 566: 40-48

**Gao Y, Woo KC** (1996) Regulation of phosphoenolpyruvate carboxylase in Zea mays by protein phosphorylation and metabolites and their roles in photosynthesis. Aust J Plant Physiol 23: 25-32

**Garcia de Frutos P, Baanante IV** (1995) The muscle isoform of 6-phosphofructo 2-kinase/fructose 2,6-bisphosphatase of the teleost Sparas aurata: relationship with the liver isoform. Arch. Biochem. Biophys. 321: 297–302

**Gardemann A** (1983) Control of CO2 fixation: regulation of spinach ribulose-5-phosphate kinase by stromal metabolite levels. Biochim Biophys Acta 722, 51–60

**Ghosh HP, Preiss J** (1966) Adenosine diphosphate glucose pyrophosphorylase. A regulatory enzyme in the biosynthesis of starch in spinach leaf chloroplasts. J Biol Chem 241: 4491-4504

**Giersch C, Heber U, Kaiser G, Walker DA, Robinson SP** (1980a) Intracellular metabolite gradients and flow of carbon during photosynthesis of leaf protoplasts. Arch Biochem Biophys 205: 246-259

**Giersch C, Heber U, Kobayashi Y, Inoue Y, Shibata K, Heldt HW** (1980b) Energy charge, phosphorylation potential and proton motive force in chloroplasts. Biochim Biophys Acta 590: 59-73

**Green DE, Leloir LF, Nocito, V** (1945) Transaminases J. Biol. Chem.; 161, 559.

**Gozzer C, Zanetti G, Galliano M, Sacchi GA, Minchiotti L, Curti B** (1977) Molecular heterogeneity of ferredoxin-NADP+ reductase from spinach leaves. Biochim Biophys Acta 485: 278-290

**Gustafson GL, Gander JE** (1972) Uridine diphosphate glucose pyrophosphorylase from Sorghum vulgare. Purification and kinetic properties. J Biol Chem 247: 1387-1397

**Hansen RG, Albrecht GJ, Bass ST, Seifert LL** (1966) Methods Enzymol.; 8, 248

**Harary I, Korey SR, Ochoa S** (1953) Biosynthesis of dicarboxylic acids by carbon dioxide fixation. VII. Equilibrium of malic enzyme reaction. J Biol Chem 203: 595-604

**Harbron S, Foyer C, Walker D** (1981) The purification and properties of sucrose-phosphate synthetase from spinach leaves: the involvement of this enzyme and fructose bisphosphatase in the regulation of sucrose biosynthesis. Arch Biochem Biophys 212: 237-246

**Hatch MD** (1987) C-4 Photosynthesis - a Unique Blend of Modified Biochemistry, Anatomy and Ultrastructure. Biochim Biophys Acta 895: 81-106

**Hatch MD, Burnell JN** (1990) Carbonic-Anhydrase Activity in Leaves and Its Role in the First Step of C-4 Photosynthesis. Plant Physiol 93: 825-828

**Hatch MD, Droscher L, Flugge UI, Heldt HW** (1984) A Specific Translocator for Oxaloacetate Transport in Chloroplasts. Febs Lett 178: 15-19

**Hawker JS, Ozbun JL, Ozaki H, Greenberg E, Preiss J** (1974) Interaction of spinach leaf adenosine diphosphate glucose alpha-1,4-glucan alpha-4-glucosyl transferase and alpha-1,4-glucan, alpha-1,4-glucan-6-glycosyl transferase in synthesis of branched alpha-glucan. Arch Biochem Biophys 160: 530-551

**Hiraga K, Kikuchi G** (1980) The mitochondrial glycine cleavage system. Purification and properties of glycine decarboxylase from chicken liver mitochondria. J Biol Chem 255: 11664-11670

**Hoofd LJC, Tong RR, Stroeve P** (1986) Nonequilibrium Facilitated Transport Of Carbon-Dioxide In Bicarbonate And Bovine Albumin Solutions. Ann Biomed Eng 14: 493-511

**Howitz KT, McCarty RE** (1985a) Kinetic characteristics of the chloroplast envelope glycolate transporter. Biochemistry 24:2645–52

**Howitz KT, McCarty RE** (1985b) Substrate specificity of the pea chloroplast glycolate transporter. Biochemistry 24: 3645–3650

**Howitz KT, McCarty RE** (1986) D-Glycerate transport by the pea chloroplast glycolate carrier. Studies on [1-14C]D-glycerate uptake and D-glycerate dependent O2 evolution. Plant Physiol 80: 390–395

**Ireland RJ, Joy KW** (1983) Purification and properties of an asparagine aminotransferase from Pisum sativum leaves. Archives of Biochemistry and Biophysics, 223, 291–296

**Iwaki T, Wadano A, Yokota A, Himeno M** (1991) Aldolase - an important enzyme in controlling the ribulose-l,5-bisphos- phate regeneration rate in photosynthesis. Plant Cell Physiol. 32, 1083-1091

**Jang HK, Lee SW, Lee YH, Hahn TR** (2003) Purification and characterization of a recombinant pea cytoplasmic fructose-1,6-bisphosphatase. Protein Expr Purif 28: 42-48

**Jenkins CL, Burnell JN, Hatch MD** (1987) Form of inorganic carbon involved as a product and as an inhibitor of c(4) Acid decarboxylases operating in c(4) photosynthesis. Plant Physiol 85: 952-957

**Jenkins CL Hatch MD** (1985) Properties and reaction mechanism of C4 leaf pyruvate,Pi dikinase. Arch Biochem Biophys 239: 53-62

**Jenkins CL, Furbank RT, Hatch MD.** (1989) Mechanism of C4 Photosynthesis - a Model Describing the Inorganic Carbon Pool in Bundle Sheath-Cells. Plant Physiol 91: 1372-1381

**Julliard JH, Breton-Gilet A** (1997) Identification of hydroxypyruvate reductase from parsley by peptide sequence comparison after a two-step purification. Protein Expr Purif 9: 10-14

**Kagawa T, Bruno PL** (1988) NADP-malate dehydrogenase from leaves of Zea mays: purification and physical, chemical, and kinetic properties. Arch Biochem Biophys 260: 674-695

**Keirns JJ, Wang JH** (1972) Studies on nicotinamide adenine dinucleotide phosphate reductase of spinach chloroplasts. J Biol Chem 247: 7374-7382

**Kerr MW, Gear CF** (1974) Phosphoglycolate phosphatase isolated from pea leaves. Biochem. Soc. Trans. 2, 338-340

**Kim Y, Yakunin AF, Kuznetsova E, Xu X, Pennycooke M, Gu J, Cheung F, Proudfoot M, Arrowsmith CH, Joachimiak A, Edwards AM, Christendat D** (2004) Structure- and function-based characterization of a new phosphoglycolate phosphatase from Thermoplasma acidophilum. J Biol Chem 279: 517-526

**Kleczkowski LA, Edwards GE** (1989) Identification of hydroxypyruvate and glyoxylate reductases in maize leaves. Plant Physiol 91: 278-286

**Kleczkowski LA, Randall DD** (1988) Purification and characterization of a novel NADPH(NADH)-dependent hydroxypyruvate reductase from spinach leaves. Comparison of immunological properties of leaf hydroxypyruvate reductases. Biochem J 250: 145-152

**Kleczkowski LA, Randall DD, Blevins DG** (1986) Purification and characterization of a novel NADPH(NADH)-dependent glyoxylate reductase from spinach leaves. Comparison of immunological properties of leaf glyoxylate reductase and hydroxypyruvate reductase. Biochem J 239: 653-659

**Kleczkowski LA, Randall DD, Zahler WL** (1985) The substrate specificity, kinetics, and mechanism of glycerate-3-kinase from spinach leaves. Arch Biochem Biophys 236: 185-194

Kleczkowski, L. A., Villand, P., Lonneborg, A., Olsen, O. A. and Luthi, E. (1991). Plant ADP-glucose pyrophosphorylase--recent advances and biotechnological perspectives (a review). Z Naturforsch C 46: 605-612

**Knaff DB** (1996) Ferredoxin and ferredoxin-dependent enzymes. In DR Ort and CF Yocum, eds, Oxygenic Photosynthesis: The Light Reactions. Kluwer Academic Publishers, Dordrecht, The Netherlands, pp 333-361

**Kochi H, Kikuchi G** (1974) Mechanism of the reversible glycine cleavage reaction in Arthrobacter globiformis. I. Purification and function of protein components required for the reaction. J Biochem 75: 1113-1127

**Kanai R, Edwards GE** (1999) The Biochemistry of C4 Photosynthesis. In RF Sage, RK Monson, eds, C4 Plant Biology. Academic Press, Toronto, pp 173–211.

**Krebs HA** (1953) Equilibria in transamination systems. Biochem. J.; 54, 82

**Kretschmer M, Hofmann E** (1984) Inhibition of rat liver phosphofructokinase-2 by phosphoenolpyruvate and ADP. Biochem Biophys Res Commun 124: 793-796

**Kruger I, Schnarrenberger C** (1983) Purification, subunit structure and immunological comparison of fructose-bisphosphate aldolases from spinach and corn leaves. Eur J Biochem 136: 101-106

**Laisk A, Eichelmann H, Oja V, Eatherall A, Walker DA** (1989) A mathematical model of the carbon metabolism in photosynthesis. Difficulties in explaining oscillations by fructose 2,6-bisphosphate regulation. Proc R Soc Lond B Biol Sci 237: 389–415

**Laisk A, Edwards GE** (2000) A mathematical model of C-4 photosynthesis: The mechanism of concentrating CO2 in NADP-malic enzyme type species. Photosynth Res 66: 199-224

**Larondelle Y, Mertens E, Van Schaftingen E, Hers HG** (1986) Purification and properties of spinach leaf phosphofructokinase 2/fructose 2,6-bisphosphatase. Eur J Biochem 161: 351-357

**Lawson JWR, Guynn RW, Cornell N, Veech RL** (1976) in "Gluconeogenesis: Its Regulation in Mammalian Species"; R.W. Hanson and M.A. Mehlman, eds.; John Wiley and Sons, New York. pp. 481-512.

**Lazova GN, Stemler AJ** (2008) A 160 kDa protein with carbonic anhydrase activity is complexed with rubisco on the outer surface of thylakoids. Cell Biol Int 32: 646-653

**Leegood RC** (1985) The Intercellular Compartmentation of Metabolites in Leaves of Zea-Mays-L. Planta 164: 163-171

**Lemaire M, Miginiac-Maslow M, Decottignies P** (1996) The catalytic site of chloroplastic NADP-dependent malate dehydrogenase contains a His/Asp pair. Eur J Biochem 236: 947-952

**Li L, Preiss J** (1992) Characterization of ADPglucose pyrophosphorylase from a starch-deficient mutant of Arabidopsis thaliana(L). Carbohydr Res 227: 227-239

**Lunn JE, ap Rees T** (1990) Apparent equilibrium constant and mass-action ratio for sucrose-phosphate synthase in seeds of Pisum sativum. Biochem J 267: 739-743

**Lunn JE, Ashton AR, Hatch MD, Heldt HW** (2000) Purification, molecular cloning, and sequence analysis of sucrose-6F-phosphate phosphohydrolase from plants. Proc Natl Acad Sci U S A 97: 12914-12919

**Macdonald FD, Chou Q, Buchanan BB, Stitt M** (1989) Purification and characterization of fructose-2,6-bisphosphatase, a substrate-specific cytosolic enzyme from leaves. J Biol Chem 264: 5540-5544

**Macioszek J, Anderson LE** (1987) Changing kinetic properties of the two enzymes phosphoglycerate kinase/NADP-linked glyceraldehyde-3-phosphate dehydrogenase couple from pea chloroplasts during photosynthetic induction. Biochem. Biophys. Acta 892:185–190.

**Markham JE, Kruger NJ** (2002) Kinetic properties of bifunctional 6-phosphofructo -2-kinase/fructose-2,6-bisphosphatase from spinach leaves. Eur J Biochem 269: 1267-1277

**McGuire M, Carroll LJ, Yankie L, Thrall SH, DunawayMariano D, Herzberg O, Jayaram B, Haley BH** (1996) Determination of the nucleotide binding site within Clostridium symbiosum pyruvate phosphate dikinase by photoaffinity labeling, site-directed mutagenesis, and structural analysis. Biochemistry-Us 35: 8544-8552

**Moorhead GB, Plaxton WC** (1990) Purification and characterization of cytosolic aldolase from carrot storage root. Biochem J 269: 133-139

**Mukerji, SK** (1977) Corn Leaf Phosphoenolpyruvate Carboxylases - Purification And Properties Of 2 Isoenzymes. Arch Biochem Biophys 182: 343-351

**Nakamura, Y, Tolbert, NE** (1983) Serine: glyoxylate, alanine:glyoxylate, and glutamate:glyoxylate aminotransferase reactions in peroxisomes from spinach leaves. J Biol Chem 258: 7631-7638

**Nakano K, Omura Y, Tagaya M, Fukui T** (1989) UDP-glucose pyrophosphorylase from potato tuber: purification and characterization. J Biochem 106: 528-532

**Nilsson U, Hecquet L, Gefflaut T, Guerard C, Schneider G** (1998) Asp477 is a determinant of the enantioselectivity in yeast transketolase. Febs Lett 424: 49-52

**O'Leary MH, Reife JE, Slater JD** (1981) Kinetic and isotope effect studies of maize phosphenolpyruvate carboxylase. Biochemistry 20:73 08--14

**O'Leary MH** (1984) Measurement of the isotopic fractionation associated with diffusion of carbon dioxide in aqueous solution. J. Phys. Chem. 88:823-25

**Omnaas J, Porter MA, Hartman FC** (1985) Evidence for a reactive cysteine at the nucleotide binding site of spinach ribulose-5-phosphate kinase. Arch Biochem Biophys 236: 646-653

**Paszkowski A, Niedzielska A** (1989) Glutamate:glyoxylate aminotransferase from the seedlings of rye (Secale cereale L.). Acta biochimica Polonica 36: 17-29

**Paszkowski, A, Niedzielska A** (1990) Serine:glyoxylate aminotransferase from the seedlings of rye (Secale cereale L.). Acta biochimica Polonica 37: 277-282

**Penefsky HS** (1974) Mitochondrial and chloroplast ATPases. In Boyer, PD (ed.) The Enzymes. Academic Press, New York, pp. 375–395.

**Pocker Y, Miksch RR** (1978) Plant Carbonic-Anhydrase - Properties And Bicarbonate Dehydration Kinetics. Biochemistry-Us 17: 1119-1125

**Porter MA, Milanez S, Stringer CD, Hartman FC** (1986) Purification and characterization of ribulose-5-phosphate kinase from spinach. Arch Biochem Biophys 245: 14-23

**Reger BJ, Ku MSB, Potter JW, Evans JJ** (1983) Purification and Characterization of Maize Ribulose-1,5-Bisphosphate Carboxylase. Phytochemistry 22: 1127-1132

**Reichert A, Baalmann E, Vetter S, Backhausen JE, Scheibe R** (2000) Activation properties of the redox-modulated chloroplast enzymes glyceraldehyde 3-phosphate dehydrogenase and fructose-1,6-bisphosphatase. Physiol. Plant. 110, 330-341

**Roeske CA, O'leary MH** (1984) Carbon isotope effects on the enzymecatalyzed carboxylation of ribulose bisphosphate. Biochemistry 23:6275-84

**Salerno GL, Pontis HG** (1978) Studies on sucrose phosphate synthetase. The inhibitory action of sucrose. Febs Lett 86: 263-267

**Schenk G, Duggleby RG, Nixon PF** (1998) Properties and functions of the thiamin diphosphate dependent enzyme transketolase. The international journal of biochemistry & cell biology 30: 1297-1318

**Schimkat D, Heineke D, Heldt HW** (1990) Regulation of sedoheptulose-1,7-bisphosphatase by sedoheptulose-7-phosphate and glycerate, and of fructose-1,6-bisphosphatase by glycerate in spinach chloroplasts. Planta 181: 97–103

**Schnarrenberger C, Kruger I** (1986) Distinction between Cytosol and Chloroplast Fructose -Bisphosphate Aldolases from Pea, Wheat, and Corn Leaves. Plant Physiol 80: 301-304

**Slabas AR, Walker DA** (1976) Inhibition of spinach phosphoribulokinase by DL-glyceraldehyde. Biochem J 153: 613-619

**Shin M** (1972) Ferredoxin-NADPH reductase from spinach. Methods Enzymol. 23:440-445.

**Son D, Jo J, Sugiyama T** (1991) Purification and characterization of alanine aminotransferase from Panicum miliaceum leaves. Arch. Biochem. Biophys. 289, 262-266

**Sonnewald U, Quick WP, MacRae E, Krause KP, Stitt M** (1993) Purification, cloning and expression of spinach leaf sucrose-phosphate synthase in Escherichia coli. Planta 189: 174-181

**Sowinski P, Szczepanik J, Minchin PEH** (2008) On the mechanism of C-4 photosynthesis intermediate exchange between Kranz mesophyll and bundle sheath cells in grasses. J Exp Bot 59: 1137-1147

**Sowokinos JR** (1981) Pyrophosphorylases in Solanum tuberosum: II. CATALYTIC PROPERTIES AND REGULATION OF ADP-GLUCOSE AND UDP-GLUCOSE PYROPHOSPHORYLASE ACTIVITIES IN POTATOES. Plant Physiol 68: 924-929

**Sowokinos JR, Spychalla JP, Desborough SL** (1993) Pyrophosphorylases in Solanum tuberosum (IV. Purification, Tissue Localization, and Physicochemical Properties of UDP-Glucose Pyrophosphorylase). Plant Physiol 101: 1073-1080

**Sparla F, Fermani S, Falini G, Zaffagnini M, Ripamonti A, Sabatino P, Pupillo P, Trost P** (2004) Coenzyme site-directed mutants of photosynthetic A4-GAPDH show selectively reduced NADPH-dependent catalysis, similar to regulatory AB-GAPDH inhibited by oxidized thioredoxin. J Mol Biol 340: 1025-1037

**Sparla F, Zaffagnini M, Wedel N, Scheibe R, Pupillo P, Trost P** (2005) Regulation of photosynthetic GAPDH dissected by mutants. Plant Physiol 138: 2210-2219

**Speranza ML, Ferri G**(1982) Glyceraldehyde-3-phosphate dehydrogenase (glycolytic form) from spinach leaves. Methods Enzymol. 89, 316-319

**Sprenger GA, Schorken U, Sprenger G Sahm H** (1995) Transketolase A of Escherichia coli K12. Purification and properties of the enzyme from recombinant strains. Eur J Biochem 230: 525-532

**Stitt M, Heldt HW** (1985) Generation and Maintenance of Concentration Gradients between the Mesophyll and Bundle Sheath in Maize Leaves. Biochim Biophys Acta 808: 400-414

**Surek B, Heilbron A, Austen A Latzko E** (1985) Purification and characterization of phosphoribulokinase from wheat leaves. Planta 165: 507-512

**Tang GL, Wang YF, Bao JS Chen HB** (2000) Overexpression in Escherichia coli and characterization of the chloroplast fructose-1,6-bisphosphatase from wheat. Protein Expr Purif 19: 411-418

**Taniguchi M, Kobe A, Kato M, Sugiyama T** (1995) Aspartate aminotransferase isozymes in Panicum miliaceum L., an NAD-malic enzyme-type C4 plant: comparison of enzymatic properties primary structures, and expression patterns. Arch. Biochem. Biophys. 318, 295-306

**Teige M, Melzer M, Suss KH** (1989). Purification, properties and in situ localization of the amphibolic enzymes D-ribulose 5-phosphate 3-epimerase and transketolase from spinach chloroplasts. Eur. J. Biochem. 252, 237-244

**Tolbert NE** (1981) Metabolic pathways in peroxisomes and glyoxysomes. Annu Rev Biochem 50: 133-157

**Trost P, Scagliarini S, Valenti V, Pupillo P** (1993) Activation of spinach chloroplast glyceraldehyde 3-phosphate dehydrogenase: Effect of glycerate 1,3-bisphosphate. Planta 190: 320–326

**Uchida K, Mochizuki M, Niizeki K** (1983) Diffusion-Coefficients Of Co2 Molecule And Bicarbonate Ion In Hemoglobin Solution Measured by Fluorescence Technique. Jpn J Physiol 33: 619-634

**Uedan, K, Sugiyama T** (1976) Purification And Characterization Of Phosphoenolpyruvate Carboxylase From Maize Leaves. Plant Physiol 57: 906-910

**Uehlein N, Otto B, Hanson DT, Fischer M, McDowell N, Kaldenhoff R** (2008) Function of Nicotiana tabacum aquaporins as chloroplast gas pores challenges the concept of membrane CO2 permeability. Plant Cell 20: 648-657

**Van RC, Pan YJ, Hsu SH, Huang YT, Hsiao YY, Pan RL** (2005) Role of transmembrane segment 5 of the plant vacuolar H+-pyrophosphatase. Biochim Biophys Acta 1709: 84-94

**Villadsen D, Nielsen TH** (2001) N-terminal truncation affects the kinetics and structure of fructose-6-phosphate 2-kinase/fructose-2,6-bisphosphatase from Arabidopsis thaliana. Biochem J 359: 591-597

**von Caemmerer S** (2000) Biochemical models of leaf photosynthesis. CSIRO Publishing, Collingwood.

**von Caemmerer S, Furbank RT** (2003) The C-4 pathway: an efficient CO2 pump. Photosynth Res 77: 191-207

**Walker GH, Huber SC** (1987) ATP-dependent activation of a new form of spinach leaf 6-phosphofructo-2-kinase/fructose 2,6-bisphosphatase. Arch Biochem Biophys 258: 58-64

**Whitaker DP** (1984) Puriﬁcation and properties of sucrose-6-phosphatase from Pisum sativum shoots. Phytochemistry 23, 2429–2430.

**Winter H, Robinson DG, Heldt HW** (1993) Subcellular Volumes And Metabolite Concentrations In Barley Leaves. Planta 191: 180-190

**Woodrow IE, Walker DA** (1983) Regulation of stromal sedoheptulose 1,7-bis- phosphatase activity and its role in controlling the reductive pentose phosphate pathway of photosynthesis. Biochim Biophys Acta 722:508-516.

**Woodrow IE, Mott KA** (1993) Modeling C3 photosynthesis—a sensitivity analysis of the photosynthetic carbon reduction cycle. Planta 191: 421–432

**Xu W, Ahmed S, Moriyama H, Chollet R** (2006) The importance of the strictly conserved, C-terminal glycine residue in phosphoenolpyruvate carboxylase for overall catalysis: mutagenesis and truncation of GLY-961 in the sorghum C4 leaf isoform. J Biol Chem 281: 17238-17245

**Yu S, Xia D, Luo Q, Cheng Y, Takano T, Liu S** (2007) Purification and characterization of carbonic anhydrase of rice (Oryza sativa L.) expressed in Escherichia coli. Protein Expr Purif 52: 379-383

**Zelitch I** (1955). Glycolic acid oxidase and glyoxylic acid reductase. In SP Colowick, NO Kaplan, eds, Methods in Enzymology, Vol I. Academic Press, New York-London, pp 528-535

**Zhu X G, De Sturler E, Long SP** (2007) Optimizing the distribution of resources between enzymes of carbon metabolism can dramatically increase photosynthetic rate: A numerical simulation using an evolutionary algorithm. Plant Physiol 145: 513-526

**Ziegler I** (1974) Malate dehydrogenase in Zea mays: properties and inhibition by sulfite. Biochim Biophys Acta 364: 28-37
